# Targeting miR-126 in inv(16) acute myeloid leukemia inhibits leukemia development and leukemia stem cell maintenance

**DOI:** 10.1038/s41467-021-26420-7

**Published:** 2021-10-22

**Authors:** Lianjun Zhang, Le Xuan Truong Nguyen, Ying-Chieh Chen, Dijiong Wu, Guerry J. Cook, Dinh Hoa Hoang, Casey J. Brewer, Xin He, Haojie Dong, Shu Li, Man Li, Dandan Zhao, Jing Qi, Wei-Kai Hua, Qi Cai, Emily Carnahan, Wei Chen, Xiwei Wu, Piotr Swiderski, Russell C. Rockne, Marcin Kortylewski, Ling Li, Bin Zhang, Guido Marcucci, Ya-Huei Kuo

**Affiliations:** 1 Gehr Family Center for Leukemia Research, Department of Hematological Malignancies Translational Science, Hematologic Malignancies and Stem Cell Transplantation Institute, Beckman Research Institute, City of Hope Medical Center, Duarte, CA 91010 USA; 2 Department of Hematology, First Affiliated Hospital of Zhejiang Chinese Medical University, Hangzhou, Zhejiang 310006 China; 3 Department of Hematology, The Second Affiliated Hospital, Zhejiang University School of Medicine, Hangzhou, Zhejiang 310009 China; 4 Integrated Genomics Core, Beckman Research Institute, City of Hope Medical Center, Duarte, CA 91010 USA; 5 Department of Molecular Medicine, Beckman Research Institute, City of Hope Medical Center, Duarte, CA 91010 USA; 6 Department of Computational and Quantitative Medicine, Division of Mathematical Oncology, Beckman Research Institute, City of Hope Medical Center, Duarte, CA 91010 USA; 7 Department of Immuno-oncology, Beckman Research Institute, City of Hope Medical Center, Duarte, CA 91010 USA

**Keywords:** Cancer stem cells, Targeted therapies, Acute myeloid leukaemia

## Abstract

Acute myeloid leukemia (AML) harboring inv(16)(p13q22) expresses high levels of miR-126. Here we show that the *CBFB-MYH11 (CM)* fusion gene upregulates miR-126 expression through aberrant miR-126 transcription and perturbed miR-126 biogenesis via the HDAC8/RAN-XPO5-RCC1 axis. Aberrant miR-126 upregulation promotes survival of leukemia-initiating progenitors and is critical for initiating and maintaining CM-driven AML. We show that miR-126 enhances MYC activity through the SPRED1/PLK2-ERK-MYC axis. Notably, genetic deletion of miR-126 significantly reduces AML rate and extends survival in CM knock-in mice. Therapeutic depletion of miR-126 with an anti-miR-126 (miRisten) inhibits AML cell survival, reduces leukemia burden and leukemia stem cell (LSC) activity in inv(16) AML murine and xenograft models. The combination of miRisten with chemotherapy further enhances the anti-leukemia and anti-LSC activity. Overall, this study provides molecular insights for the mechanism and impact of miR-126 dysregulation in leukemogenesis and highlights the potential of miR-126 depletion as a therapeutic approach for inv(16) AML.

## Abstract (web summary)

miR-126 is highly expressed in inv(16) Acute myeloid leukemia (AML) but its role is unclear. Here, the authors show that the aberrant expression of miR-126 in inv(16) AML is directly due to the *CBFB-MYH11* fusion gene and that it can promote AML development and leukemia stem cell maintenance, highlighting miR-126 as a therapeutic target for inv(16) AML patients

## Introduction

Acute myeloid leukemia (AML) is an aggressive hematopoietic malignancy characterized by excessive proliferation of immature leukemic blasts. Approximately 21,000 patients are diagnosed with AML each year in the United States, and the latest 5-year overall survival rate remains at only 28% (https://seer.cancer.gov)^1,2^. AML is maintained and propagated by a population of leukemia-initiating cells or leukemia stem cells (LSCs), which feature quiescence and therapy resistance, and contribute to subsequent clonal evolution of the disease and relapse^3,4^.

AML comprises multiple entities characterized by specific gene mutations and chromosomal abnormalities that drive leukemogenesis and can be used as prognosticators^5,6^. One of the most frequent chromosomal translocations detected in 5–12% AML patients is chromosome 16 inversion, inv(16)(p13q22) or t(16;16)(p13.1;q22) [henceforth inv(16)], which is associated with the FAB M4Eo AML subtype^7,8^. At the molecular level, inv(16) disrupts the *CBFB* gene encoding the CBFβ subunit of the core-binding factor (CBF) transcription factor complex that acts as a master regulator of hematopoietic development and lineage specification^9,10^. Specifically, inv(16) breaks and joins *CBFB* and the smooth-muscle myosin heavy chain (*MYH11*) gene, creating a fusion gene *CBFB-MYH11* (CM) which encodes a fusion protein CBFβ-SMMHC^11^. Although inv(16) AML patients have a relatively favorable prognosis, only approximately 50–60% eventually achieve long-term survival with standard chemotherapy^12,13^. Therefore, the development of therapies capable of targeting LSCs is necessary to achieve a cure in the vast majority of the patients.

MicroRNAs (miRNAs) are small non-coding RNA molecules that modulate multiple targets by promoting mRNA degradation or repressing translation^14,15^. MiRNAs are transcribed as primary (pri)-miRNAs, processed into precursor (pre)-miRNAs by DROSHA, exported into the cytoplasm via the RAN (a small GTP-binding RAS-related nuclear protein)-Exportin-5 (XPO5) complex^16,17^, and processed by Dicer into mature miRNAs^18^. In hematopoiesis, miRNAs play critical roles in coordinating context-dependent differentiation programs and cell fate decisions^19^. MiR-126 is highly expressed in endothelial cells and plays a key role in angiogenesis, vascular development and homeostasis^20,21^. Within the hematopoietic compartment, miR-126 expression is enriched in long-term hematopoietic stem cells (HSCs) and plays a pivotal role in restraining cell cycle progression and maintaining HSC quiescence^22^. Aberrant miRNA expression profiles have been associated with malignant transformation and prognosis in leukemia and other hematologic malignancies^23–27^. Notably, miR-126 is aberrantly expressed in CBF leukemias including t(8;21) and inv(16)^25,26^. We and others have reported that miR-126 contributes to LSC activity, maintenance, and drug resistance in myeloid leukemias^28–30^. Furthermore, we recently reported that activating tyrosine kinase mutations (e.g., BCR-ABL, FLT3-ITD) deregulate intronic miRNA (e.g., miR-126) biogenesis by interfering with RAN-XPO5 mediated pre-miRNA processing via phosphorylation of SPRED1, a member of the Sprouty family of proteins^30,31^.

Here, we investigated the mechanism and functional impact of miR-126 dysregulation in inv(16) AML. We utilized the conditional *Cbfb-MYH11* knock-in mouse model^32,33^ combined with a miR-126 floxed allele^34^ to determine the function and regulatory mechanism of miR-126 during inv(16)-induced AML development and evaluated the efficacy of targeting LSCs using miRisten, an anti-miR-126 oligonucleotide therapeutic.

## Results

### *Cbfb-MYH11* (*CM*) upregulates miR-126 expression in hematopoietic stem and progenitor cells (HSPCs)

**Fig. 1:**
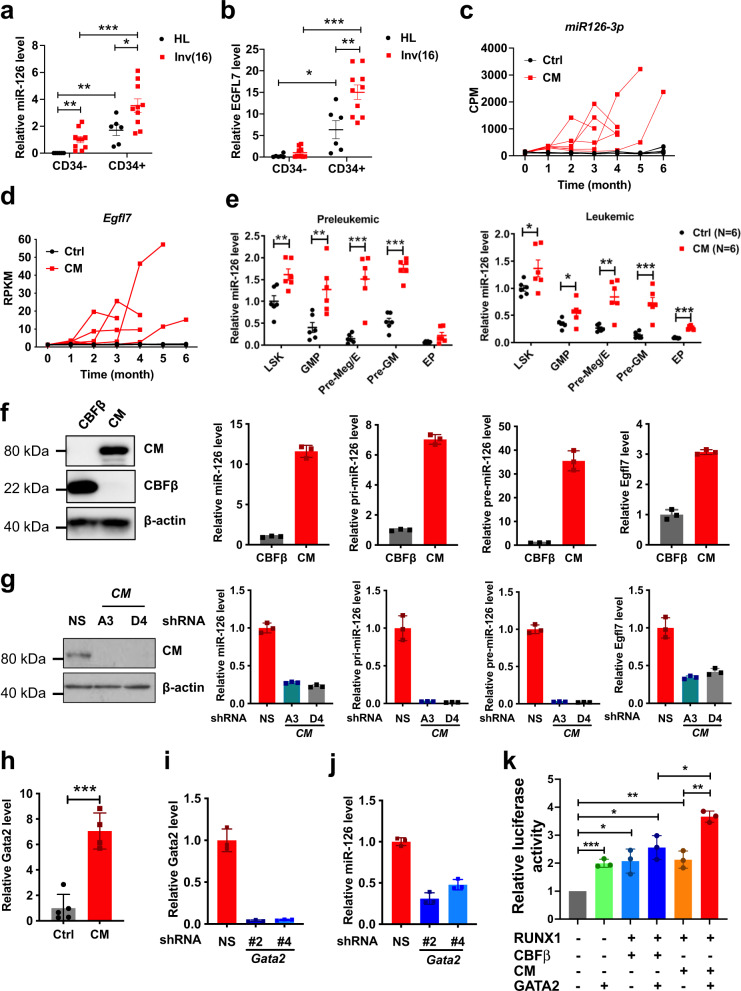
*Cbfb-MYH11 (CM)* upregulates miR-126 expression in HSPC. **a** Relative levels of miR-126 in CD34^+^ and CD34^−^ cells from healthy (HL; black circle; *n* = 6; HL CD34^+^ vs. HL CD34^−^ *p* = 0.0036) donors and inv(16) AML patients (red square; *n* = 10; inv(16) CD34^+^ vs. HL CD34^+^ *p* = 0.024; inv(16) CD34^−^ vs. HL CD34^−^ *p* = 0.0053; inv(16) CD34^+^ vs. inv(16) CD34^−^ *p* = 0.0002). **b** Relative levels of *EGFL7* in CD34^+^ and CD34^−^ cells from HL donors (black circle; *n* = 6; HL CD34^+^ vs. HL CD34^−^ *p* = 0.016) and inv(16) AML patients (red square; *n* = 10; inv(16) CD34^+^ vs. HL CD34^+^ *p* = 0.006; inv(16) CD34^+^ vs. inv(16) CD34^−^ *p* < 0.0001). **c** Counts per million (CPM) reads for miR-126-3p in PB based on miRNA-seq of control (Ctrl; black line; *n* = 7) and CM (red line; *n* = 6) mice over time until moribund with leukemia (two-way ANOVA analysis showed CM vs. Ctrl *p* < 0.0001). **d** Normalized reads per kilobase per million of transcript (RPKM) for *Egfl7* in PB based on RNA-seq of Ctrl (black line; *n* = 7) and CM (red line; *n* = 6) mice over time (two-way ANOVA analysis showed Ctrl vs. CM *p* = 0.0003). **e** Relative levels of miR-126 in CM (red square) preleukemic (6 weeks after induction; left; *n* = 6) or leukemic (right; *n* = 6) vs. Ctrl (black circle; *n* = 6) HSPC populations, including LSK (preleukemic *p* = 0.0074; leukemic *p* = 0.049), GMP (preleukemic *p* = 0.0064; leukemic *p* = 0.043), Pre-Meg/E (preleukemic *p* = 0.00015; leukemic *p* = 0.0012), Pre-GM (preleukemic *p* < 0.0001; leukemic *p* = 0.0002), and EP (leukemic *p* < 0.0001). **f** Western blot for CBFβ and CM using anti-CBFβ in 32D cells transduced with *CBFB* vs. *CM* (left). Relative levels of primary *pri-miR-126*, precursor *pre-miR-126*, mature miR-126, and *Egfl7* in 32D cells expressing CM (red dots/bars) vs. CBFβ (black dots, gray bars). **g** Western blot analysis for CM in 32D-CM non-silencing (NS) control vs. *CM* shRNA (A3, D4) cells (left). Relative levels of mature miR-126, pri-miR-126, pre-miR-126 and *Egfl7* expression in NS (red), *CM* shRNA-A3 (blue) or *CM* shRNA-D4 (gray) cells (right). **h** Relative *Gata2* expression in BM samples of control (black/gray; *n* = 5) vs. CM leukemia (red; *n* = 4; *p* = 0.0002) mice. **i** Relative level of *Gata2* in leukemia BM cells transduced with NS control (red), *Gata2* shRNA #2 (dark blue), *Gata2* shRNA #4 (light blue). **j** Relative expression of miR-126 in leukemia BM cells transduced with NS control (red), *Gata2* shRNA #2 (dark blue), *Gata2* shRNA #4 (light blue). **k** *EGFL7* promoter-firefly luciferase reporter activity normalized with renilla luciferase as internal control in 293T cells co-transfected with or without GATA2, RUNX1, CBFβ or CM expression vectors as indicated (gray: none; green: GATA2; light blue: RUNX1 + CBFβ; dark blue: RUNX1 + CBFβ + GATA2; orange: RUNX1 + CM; Red: RUNX1 + CM + GATA2). Each dot represents results from an individual sample and data are presented as the mean ± SEM in **a**, **b**, **e**, **h**; each line represents the trajectory of an individual mouse in **c**, **d**. Representative data of at least three independent experiments are shown in **f**, **g**, **i**, **j** and data are presented as the mean ± SD. Q-PCR data in **a**, **b**, **e**, **f** for *pri-miR-126*, *pre-miR-126*, *Egfl7*, and *Gata2* were normalized to Beta-2-microglobulin (*B2M*) and miR-126 data were normalized to *RNU44* (human) or *snoRNA234* (mouse). The statistical significance for all comparisons shown was determined using two-tailed Student’s *T* tests (*p < 0.05; **p < 0.01; ***p < 0.001).

Previous studies have shown that miR-126 is highly expressed and contributes to the relative quiescence of normal CD34^+^ HSCs^22,28–30^. Herein we show that CD34^+^ cells from inv(16) AML patients have even higher miR-126 levels than CD34^+^ cells from normal healthy donors (Fig. 1a). MiR-126 is located within intron 7 of the *EGFL7* gene^35^, which was highly co-expressed with miR-126 (Fig. 1b).

We previously generated a conditional *CM* knock-in (*Cbfb*^*56M/+*^*/Mx1-Cre*) mouse model^32,33^, which recapitulates human inv(16) AML development in an average of 3–4 months after induction of CM expression by poly(I:C) treatment. In a time-sequential analysis of miR-126 levels in mononuclear cells from peripheral blood (PB) of *CM* knock-in mice, we observed a progressive increase of both miR-126 and *Egfl7* levels over time (Fig. 1c, d) and in multiple CM HSPC populations^33,36,37^ (Fig. 1e). Significant upregulation of miR-126 was detected as early as 1 month after CM induction (Supplementary Fig. 1a).

### CM upregulates *Egfl7/miR-126* transcription in concert with Gata2

To determine whether CM directly dysregulates miR-126 expression, we forced CM or wild-type CBFβ expression in 32D myeloblast cells via MSCV-IRES-GFP (MIG) retroviral transduction. We observed upregulated miR-126, *pri-miR-126* and *pre-miR-126* as well as *Egfl7* in *CM*-transduced GFP^+^ cells compared to *CBFB*-transduced GFP^+^ cells (Fig. 1f). Conversely, shRNA-mediated CM knockdown resulted in significant downregulation of miR-126, *pri-miR126*, *pre-miR-126* and *Egfl7* in 32D-CM cells (Fig. 1g). We and others have shown that CM induces upregulation of the *Gata2* transcription factor^33,38^, which reportedly transactivates the *EGFL7*/*miR-126* promoter^39,40^. Accordingly, we observed increased *Gata2* levels upon CM transduction in 32D cells (Supplementary Fig. 1b), as well as in *CM* knock-in PB (Supplementary Fig. 1c) along with miR-126/*Egfl7* levels (Fig. 1c, d), and AML cells from *CM* knock-in mice (Fig. 1h). Conversely, *Gata2* knockdown in *CM* knock-in AML cells resulted in downregulation of miR-126 (Fig. 1I, j) and *Egfl7* (Supplementary Fig. 1d). Analysis of the promoter regions of both murine and human *EGFL7* identified several putative CBF binding sites (TRANSFAC database^41^) nearby the GATA-binding sites (Supplementary Fig. 1e). Chromatin immunoprecipitation (ChIP)-qPCR showed that both CBFβ and CM protein indeed could occupy these CBF sites within the *Egfl7* promoter region and that some of the nearby GATA sites were also occupied by GATA2 (Supplementary Fig. 1e). In addition, we confirmed RUNX1, CBFβ/CM occupancy on the reported RUNX1 site^42,43^ as well as GATA2 binding to the reported GATA2 site^42,43^ in the human *EGFL7* promoter by ChIP-qPCR using primary inv(16) AML CD34^+^ cells (Supplementary Fig. 1f). We then performed luciferase reporter assays to evaluate transactivation of the human *EGFL7* promoter^44^ by GATA2 and the CBF complex, which is consisted of RUNX1/CBFβ or RUNX1/CM. A significant increase of *EGFL7-luciferase* transactivation was observed with forced expression of GATA2, RUNX1/CBFβ and RUNX1/CM (Fig. 1k). Co-transfection of RUNX1/CBFβ or RUNX1/CM with GATA2 further increased *EGFL7* promoter transactivation, suggesting a co-regulatory activity of GATA2 and RUNX1/CBF on *EGFL7*/*miR-126* transcription.

### CM increases biogenesis of mature miR-126 through the HDAC8/RAN–XPO5–RCC1 axis

**Fig. 2:**
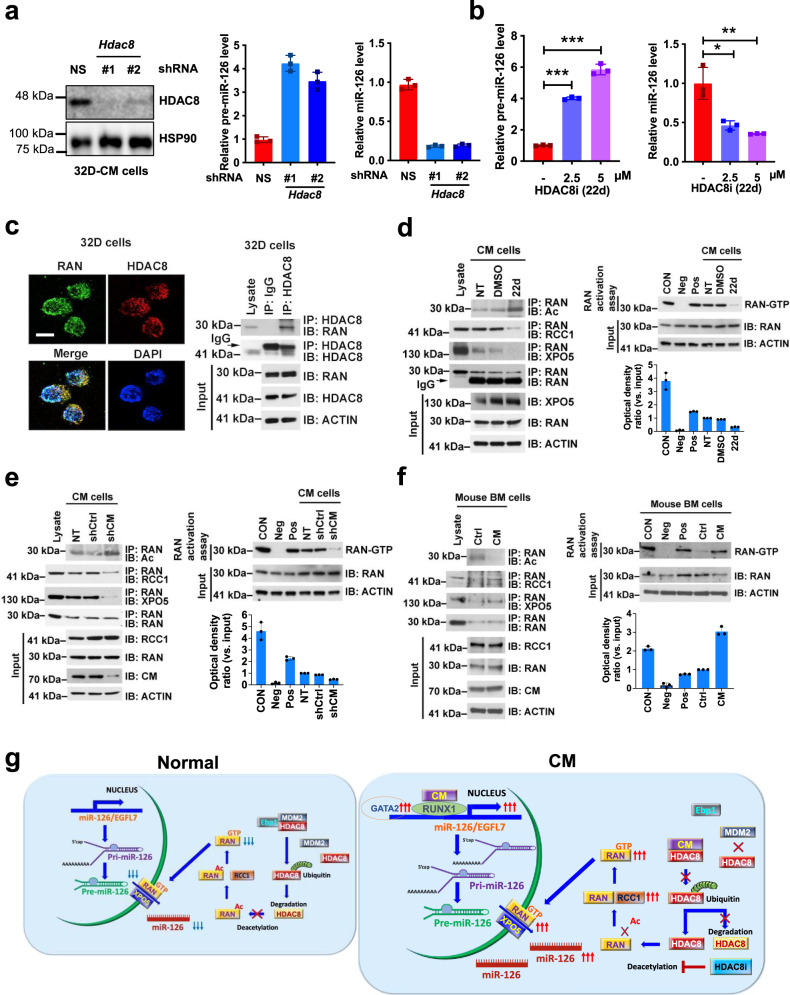
CM upregulates miR-126 biogenesis through HDAC8/RAN-XPO5 axis. **a** Western blot analysis of HDAC8 in 32D-CM cells with NS or *Hdac8* shRNA (#1, #2) (left). Relative expression of *pre-miR-126* and mature miR-126 in 32D-CM cells with NS (red) vs. *Hdac8* shRNA#1 (light blue), *Hdac8* shRNA#2 (dark blue) (right). **b** Relative expression in 32D-CM cells treated with vehicle (red) vs. HDAC8i (22d 2.5 μM or 5 μM; purple) for *pre-miR-126* (left; 2.5 μM *p* < 0.0001; 5 μM *p* < 0.0001) and miR-126 (right; 2.5 μM *p* = 0.012; 5 μM *p* = 0.0055). **c** Representative image of IF co-staining of RAN (green) and HDAC8 (red) in 32D cells (left; scale bar 10 μm). IP with anti-IgG control or anti-HDAC8 followed by immunoblotting (IB) with anti-RAN antibodies (right). Representative results of two independent experiments with similar results are shown. **d** IP with anti-RAN and IB with antibodies for Ac-Lys, RCC1, XPO5, or RAN in 32D-CM cells not-treated (NT) or treated with DMSO vehicle or 22d (2 µM) (left). The levels of RAN-GTP determined by RAN activation assay (right). The densitometry of RAN-GTP levels measured from three assays are shown on the bottom. **e** IP with anti-RAN and IB with antibodies for Ac-Lys, RCC1, XPO5, or RAN (left), and RAN activation assay (right) in 32D-CM cells not-treated (NT) or transduced with shCtrl control or shCM for 24 h. **f** IP with anti-RAN and IB with antibodies for Ac-Lys, RCC1, XPO5, or RAN (left), and RAN activation assay (right) of BM cells isolated from Ctrl or CM mice. **g** Schematic model of the mechanism by which CM regulates *miR-126/EGFL7* transcription as well as miR-126 biogenesis through enhancing HDAC8 activity which in turn promotes RAN-XPO5 mediated transportation. *Pre-miR-126* was normalized to *B2m* and mature miR-126 was normalized to *snoRNA234*. Data are presented as the mean ± SD and statistical significance shown was determined using two-tailed Student’s *T* tests (*p < 0.05; **p < 0.01; ***p < 0.001).

Next, we tested whether additional mechanisms other than transcriptional regulation of *EGFL7*/*miR-126* were active in inducing high levels of mature miR-126 in CM cells. To this end, we previously reported and confirmed that CM interacts and enhances the activity of a class I histone deacetylase (HDAC), HDAC8^45^ (Supplementary Fig. 2a). Notably, HDAC8 overexpression (OE) in 32D cells significantly increased mature miR-126 levels while reducing the *pre-miR-126* precursor levels compared to vector alone (Supplementary Fig. 2b). Conversely, *Hdac8* knockdown or treatment with an HDAC8-selective inhibitor (HDAC8i; 22d) significantly downregulated the levels of mature miR-126 and increased those of pre-miR-126 (Fig. 2a, b). These results led us to postulate a possible regulatory role of CM in enhancing the miR-126 biogenesis via HDAC8.

First, we tested the impact of CM on HDAC8 protein stability. We found that CM expression enhanced the half-life of HDAC8 protein following cycloheximide treatment (Supplementary Fig. 2c). HDAC8 protein is subjected to MDM2-mediated ubiquitination (Supplementary Fig. 2d), which was reduced by CM expression (Supplementary Fig. 2e, left) and increased by CM knockdown (Supplementary Fig. 2e, right). ErbB3-binding protein 1 (EBP1) reportedly interacts with another HDAC family member HDAC2^46,47^. We showed that EBP1 participates in the regulation of HDAC8 ubiquitination as supported by co-localization and physical interaction of HDAC8 and EBP1 in 32D cells (Supplementary Fig. 2f) and reduced MDM2-induced HDAC8 ubiquitination upon EBP1 knockdown (Supplementary Fig. 2g). Forced CM expression significantly decreased the EBP1-HDAC8 interaction (Supplementary Fig. 2h, left, top panel), reduced HDAC8 ubiquitination (Supplementary Fig. 2e, left), and enhanced HDAC8 stability (Supplementary Fig. 2h, left, bottom panels). Conversely, CM knockdown rescued these effects (Supplementary Fig. 2e, h, right). Taken altogether, these results support the notion that CM prevents HDAC8-EBP1 binding thereby stabilizing and protecting HDAC8 protein from degradation.

In canonical miRNA biogenesis, nucleus-to-cytoplasm shuttling and maturation of miRNAs are mediated by a protein complex comprising RAN, XPO5, and regulator of chromosome condensation 1 (RCC1)^16,48^. We previously reported that *pre-miR-126* transport is mediated by the activity of the RAN–XPO5–RCC1 complex^30^. Utilizing immunofluorescence (IF) and IP assays, we observed colocalization and interaction of HDAC8 with RAN (Fig. 2c). Acetylation of RAN protein reportedly interferes with RAN–RCC1 binding, RAN-GTP/GDP cycles, and RAN function in nucleus–cytoplasm transport^49^. Thus, we hypothesized that the interaction of HDAC8 with RAN leads to deacetylation of RAN and enhanced activation of the nucleus–cytoplasm transportation of *pre-miR-126*, which results in high levels of mature miR-126. Consistent with our hypothesis, we showed that HDAC8 inhibition with HDAC8i (22d) treatment in 32D-CM cells led to RAN hyperacetylation (Fig. 2d, left, first panel) and decrease of RAN–RCC1 binding (Fig. 2d, left, second panel), RAN-GTP level (Fig. 2d, right), RAN–XPO5 binding (Fig. 2d, left, third panel), and mature miR-126 levels (Fig. 2b). CM knockdown in 32D-CM cells showed similar effects (Fig. 2e). Conversely, CM expression stabilized HDAC8 and in turn increased RAN deacetylation, RAN-GTP level, and RAN–XPO5–RCC1 binding in CM mouse BM cells (Fig. 2f).

Taken altogether, these results indicate that CM upregulates miR-126 expression through multiple regulatory mechanisms of miR-126 transcription and biogenesis (Fig. 2g).

### MiR-126 is critical for initiating and maintaining CM-driven AML

**Fig. 3:**
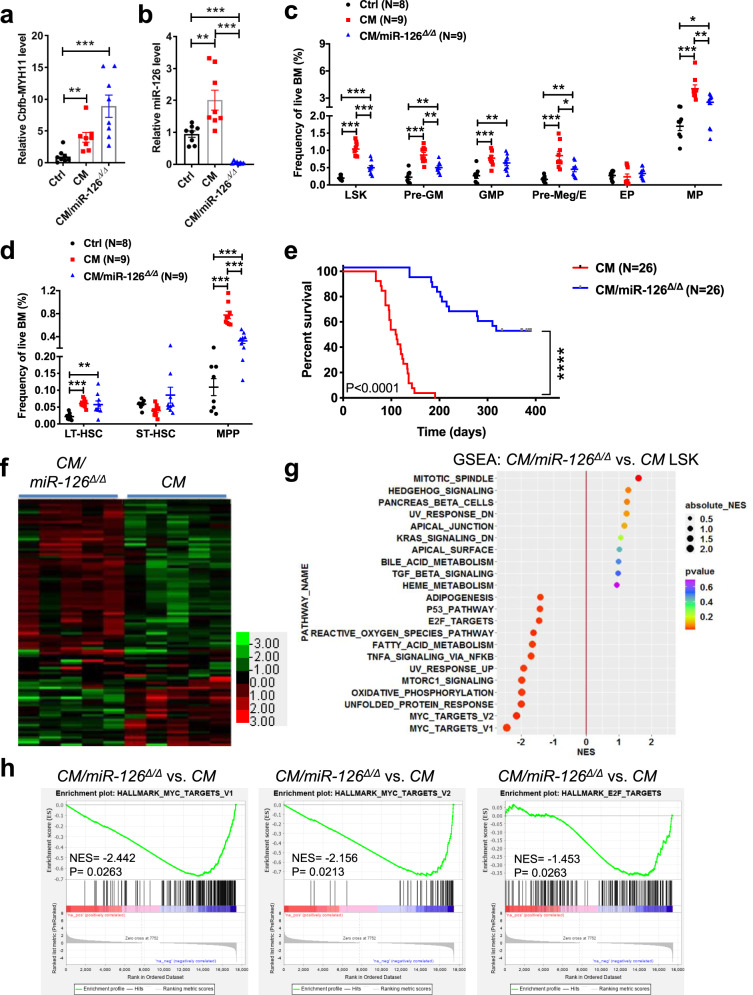
High miR-126 promotes survival of HSPCs and leukemia-initiation induced by CM. **a** Relative *Cbfb-MYH11* expression in control (Ctrl; black), *CM* (red; *CM* vs. Ctrl *p* = 0.0031), and *CM/miR-126*^*Δ/Δ*^ (blue; *CM/miR-126*^*Δ/Δ*^ vs. Ctrl *p* = 0.0006) preleukemic BM cells. **b** Relative expression levels of miR-126 in control (black), *CM* (red; *CM* vs. Ctrl *p* = 0.0062), and *CM/miR-126*^*Δ/Δ*^ (blue; *CM* vs. *CM/miR-126*^*Δ/Δ*^ *p* < 0.0001, *CM/miR-126*^*Δ/Δ*^ vs. Ctrl *p* < 0.0001) preleukemic BM cells. **c** Frequency of LSK (*CM* vs. Ctrl *p* < 0.0001; *CM/miR-126*^*Δ/Δ*^ vs. Ctrl *p* = 0.0009; *CM* vs. *CM/miR-126*^*Δ/Δ*^ *p* < 0.0001), myeloid/erythroid progenitor populations including Pre-GM (*CM* vs. Ctrl *p* < 0.0001; *CM/miR-126*^*Δ/Δ*^ vs. Ctrl *p* = 0.0046; *CM* vs. *CM/miR-126*^*Δ/Δ*^ *p* = 0.0012), GMP (*CM* vs. Ctrl *p* = 0.0002; *CM/miR-126*^*Δ/Δ*^ vs. Ctrl *p* = 0.0043), Pre-Meg/E (*CM* vs. Ctrl *p* = 0.0001; *CM/miR-126*^*Δ/Δ*^ vs. Ctrl *p* = 0.0028; *CM* vs. *CM/miR-126*^*Δ/Δ*^ *p* = 0.012), MP (*CM* vs. Ctrl *p* = 0.0001; *CM/miR-126*^*Δ/Δ*^ vs. Ctrl *p* = 0.017; *CM* vs. *CM/miR-126*^*Δ/Δ*^ *p* = 0.0079) in Ctrl (black), *CM* (red) and *CM/miR-126*^*Δ/Δ*^ (blue) preleukemic BM. Flow cytometry gating strategies for HSPC populations are shown in Supplementary Fig. 3A. **d** Frequency of LT-HSC (*CM* vs. Ctrl *p* < 0.0001; *CM/miR-126*^*Δ/Δ*^ vs. Ctrl *p* = 0.008), ST-HSC, and MPP (*CM* vs. Ctrl *p* < 0.0001; *CM/miR-126*^*Δ/Δ*^ vs. Ctrl *p* = 0.0009; *CM* vs. *CM/miR-126*^*Δ/Δ*^ *p* < 0.0001) subsets in control (black), *CM* (red), and *CM/miR-126*^*Δ/Δ*^ (blue) preleukemic BM. **e** Kaplan–Meier survival curve of induced *CM* (red line; *n* = 26; median survival 110 days) or *CM/miR-126*^*Δ/Δ*^ mice (blue line; *n* = 26; median survival 354 days) monitored up to 1 year. The statistical significance was determined using Log-rank (Mantel–Cox) test (*p* < 0.0001). **f** The hierarchical clustering heatmap is composed of log2-based RPKM values of the top 100 differential expression genes (≥1.5-fold change; *p* < 0.01) identified by RNA-seq analysis of *CM/miR-126*^*Δ/Δ*^ LSK (*n* = 5) vs. *CM* LSK (*n* = 5). The color scale is originated from the “redgreen” palette in Cluster 3.0 software, while the color intensity is related to the range of log2-based RPKM values (−3 to 3). The quasi-likelihood (QL) *F*-test (one-sided) with likelihood ratio test is carried out to determine the differentially expressed genes (DEG). The “Benjamini–Hochberg” approach is implemented for calculating adjusted *p* values in multiple comparisons. **g** Normalized enrichment score (NES) plot for top 10 most positively and negatively enriched Hallmark Signature pathways identified by GSEA. **h** Enrichment plots for most significantly enriched pathways (ordered by *p* value). The enrichment score (ES) is calculated based on the pre-ranked gene list obtained by “−log10(*p* value for DEG) × sign(log2(fold change))”. Nominal *p* value (one-sided) is estimated from background ES (derived from the random permutation of genes) by using the positive or negative portion of the distribution for the sign of ES. The adjusted significance of ES is calculated by false discovery rate (FDR) for multiple hypothesis testing. The detailed results for top 13 most enriched pathways are shown in Supplementary Table 2. Q-PCR data for *Cbfb-MYH11* were normalized with *Hprt* and miR-126 was normalized to *SnoRNA234* in **a**, **b**. Each dot in **a**–**c** represents result from an individual mouse and data are presented as the mean ± SEM; statistical significance was determined using two-tailed Student’s *T-*tests (*p < 0.05; **p < 0.01; ***p < 0.001).

To further investigate the contribution of miR-126 to CM-driven leukemogenesis in vivo, we generated mice with conditional *CM* knock-in and *miR-126* floxed alleles (*Cbfb*^*56M/+*^*/miR-126*^*flox/flox*^*/Mx1-Cre; CM/miR-126*^*Δ/Δ*^). CM expression and miR-126 deletion in *CM/miR-126*^*Δ/Δ*^ mice (Fig. 3a, b) were then induced with poly(I:C) as previously described^30,32–34^. Deletion of miR-126 alone did not alter the immunophenotype or frequencies of HSPC populations (Supplementary Fig. 3a–d). We previously reported that CM expression leads to expansion of multiple BM HSPC populations preceding the development of leukemia^32,33,50^. Deletion of miR-126 in CM mice reduced the preleukemic expansion of HSPC populations (Fig. 3c, d and Supplementary Fig. 3a) and reversed the CM-induced stress-resistance survival of leukemia-initiating progenitor populations previously defined^33^ (Supplementary Fig. 4). *CM/miR-126*^*Δ/Δ*^ mice (*n* = 26) had a significantly reduced rate of overt AML (50% vs. 100%) and a significantly extended survival (median survival 354 vs. 110 days; *p* < 0.0001) compared to *CM* mice (*n* = 26) (Fig. 3e). Transplantation of *CM* preleukemic BM cells into endothelial cell (EC)-*miR-126*^*Δ/Δ*^ or wild type (WT) recipients showed a similar rate of AML development and similar survival time (Supplementary Fig. 5), suggesting that miR-126 expression in HSPCs is critical for initiation of CM-driven AML.

**Fig. 4:**
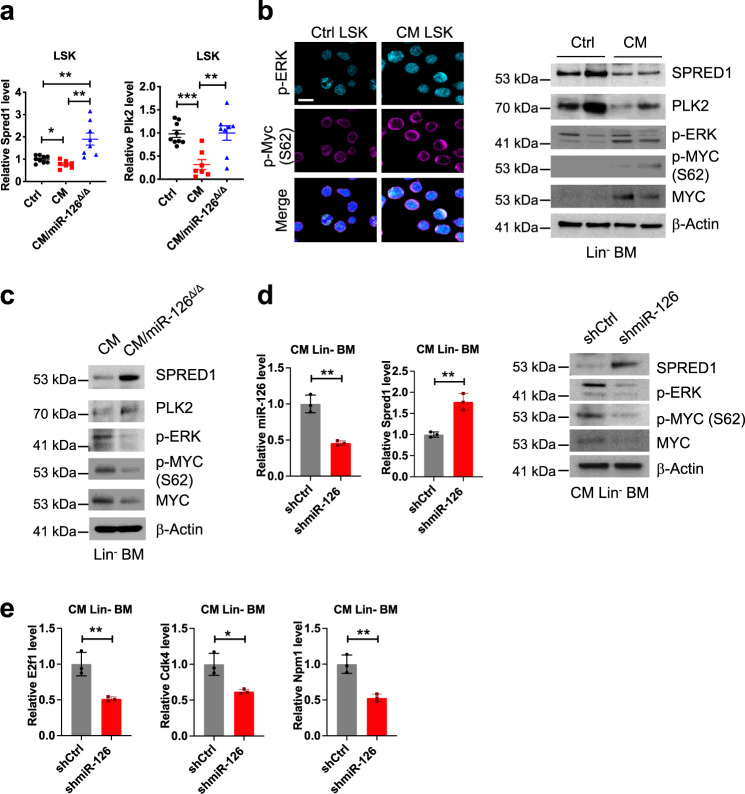
Expression of miR-126 enhances MYC activity through downregulating *SPRED1*, *PLK2*, and activating the ERK-MYC axis in CM-AML. **a** Relative expression levels *Spred1* (*CM* vs. Ctrl *p* = 0.015; *CM/miR-126*^*Δ/Δ*^ vs. Ctrl *p* = 0.0022; *CM* vs. *CM/miR-126*^*Δ/Δ*^ *p* = 0.0035) and *Plk2* (*CM* vs. Ctrl *p* = 0.0002; *CM* vs. *CM/miR-126*^*Δ/Δ*^ *p* = 0.0042) normalized to level of *B2m* in Ctrl (black), *CM* (red) and *CM/miR-126*^*Δ/Δ*^ (blue) preleukemic LSK. **b** Representative immunostaining of p-MYC (S62; magenta) and p-ERK (cyan) in control and CM AML LSK (left; scale bar 10 μm); western blot of SPRED1, PLK2, p-ERK, p-MYC (S62), and MYC in control and CM AML lineage-negative (Lin^−^) BM cells (right). **c** Western blot of SPRED1, PLK2, p-ERK, p-MYC (S62) and MYC in *CM* vs. *CM/miR-126*^*Δ/Δ*^ Lin^-^ BM cells. **d** Relative expression in CM-AML cells expressing shCtrl (black) vs. shmiR-126 (red) for miR-126 (*p* = 0.0018) and *Spred1* (*p* = 0.0028) normalized to snoRNA234 and *B2m*, respectively (left); western blot of SPRED1, p-ERK, p-MYC (S62), and MYC (right) 2 days after transduction with shCtrl or shmiR-126 (right). **e** Relative expression levels of *E2f1* (*p* = 0.0072), *Cdk4* (*p* = 0.0136), *Npm1* (*p* = 0.0041) normalized to level of *B2m* in shCtrl (black) or shmiR-126 (red) expressing CM-AML cells. Each dot represents data from an individual mouse and presented as the mean ± SEM in **a**; data in **d**, **e** are presented as the mean ± SD and statistical significance for all comparisons shown was determined using two-tailed *T* tests (*p < 0.05; **p < 0.01; ***p < 0.001); representative results of two independent experiments with similar results are shown in **b**, **c**.

To identify molecular pathways regulated by miR-126, we performed RNA-seq using sorted LSK cells isolated from BM of *CM/miR-126* ^*Δ/Δ*^ (*n* = 5) and *CM* mice (*n* = 5). We identified a total of 100 genes differentially expressed (68 genes upregulated and 32 genes downregulated) with at least 1.5-fold change (*p* < 0.01) in expression levels (Fig. 3f and Supplementary Table 1). Gene set enrichment analysis (GSEA) revealed that MYC and E2F targets, mitotic spindle, reactive oxygen species pathway, oxidative phosphorylation, TNFα signaling, and p53 pathways were differentially regulated in *CM/miR-126* ^*Δ/Δ*^ compared to *CM* LSK (Fig. 3g, h; Supplementary Fig. 6; Supplementary Table 2). SPRED1 and PLK2 (polo-like kinase 2) are previously reported miR-126 targets^21,23,25,51^ and negative regulators of RAS–MEK–ERK signaling^52,53^, which regulates MYC protein stability by phosphorylation of S62^54^. We verified that both *Spred1* and *Plk2* mRNA and protein levels are downregulated in *CM* LSK and upregulated in *CM/miR-126*^*Δ/Δ*^ LSK cells (Fig. 4a, b). Consistent with downregulation of *Spred1* and *Plk2*, CM LSK showed increased levels of phosphorylated (p)-ERK, increased p-MYC (S62) and total MYC protein (Fig. 4b). Deletion of miR-126 reversed these effects in that increased *Spred1*, *Plk2* in LSK (Fig. 4a), and decreased p-ERK, p-MYC (S62), and MYC protein levels were detected in *CM/miR-126*^*Δ/Δ*^ Lin^−^ BM cells (Fig. 4c). Similar results were obtained by knocking down miR-126 using a lentiviral vector (GFP^+^) expressing miR-126-3p shRNA (shmiR-126) (Fig. 4d). In line with downregulation of MYC level, expression of MYC target genes including *E2f1*^55^, *Cdk4*^56^, *Npm1*^57^ were significantly reduced (Fig. 4e). These results suggest that miR-126 functions to enhance MYC activity through downregulating SPRED1, PLK2, and activating the ERK–MYC axis.

**Fig. 5:**
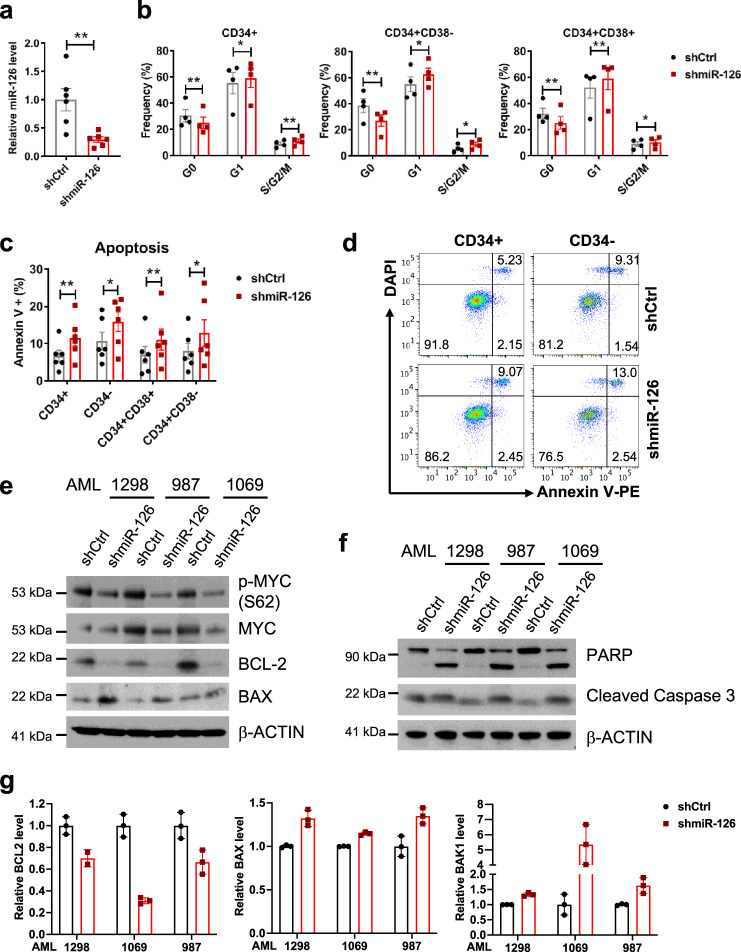
Knockdown of miR-126 promotes apoptosis and reduces quiescence of primitive inv(16) AML cells. **a** Relative levels of miR-126 in inv(16) AML CD34^+^ cells transduced with shCtrl (black) or shmiR-126 (red; *p* = 0.006), as assessed by qPCR and normalized with internal levels of RNU44. **b** Frequency (%) of G_0_, G_1_ or S/G_2_/M phases of cell cycle in shCtrl (black) or shmiR-126 (red) transduced inv(16) AML CD34^+^ (G_0_ *p* = 0.0073; G_1_ *p* = 0.046; S/G_2_/M *p* = 0.0094), CD34^+^CD38^−^ (G_0_ *p* = 0.0069; G_1_ *p* = 0.0184; S/G_2_/M *p* = 0.0138), and CD34^+^CD38^+^ (G_0_ *p* = 0.0072; G_1_ *p* = 0.0015; S/G_2_/M *p* = 0.0259) populations. **c** Frequency (%) of apoptotic cells defined by Annexin V^+^ in shCtrl (black) vs. shmiR-126 (red) transduced inv(16) AML CD34^+^ (*p* = 0.0057), CD34^−^ (*p* = 0.0261), CD34^+^CD38^+^ (*p* = 0.0027), and CD34^+^CD38^−^ (*p* = 0.0331) populations. **d** Representative FACS plots showing gating strategy and frequency of Annexin V/DAPI staining in CD34^+^ and CD34^−^ fractions of inv(16) AML samples transduced with shCtrl or shmiR-126. **e** Western blot of p-MYC (S62), MYC, BCL2, BAX in CD34^+^ cells from inv(16) AML patients (AML1298, 987, 1069) 2 days after transduction with shCtrl or shmiR-126. **f** Western blot of PARP and Cleaved Caspase 3 in CD34^+^ cells from inv(16) AML patients (AML1298, 987, 1069) 2 days after transduction of shCtrl or shmiR-126. **g** Relative levels of *BCL2*, *BAX*, *BAK1* in CD34^+^ cells from inv(16) AML patients (AML#1298, 1069, 987) transduced with shCtrl (black) or shmiR-126 (red), as assessed by qPCR and normalized using internal levels of *B2M*. Data are presented as the mean ± SD. Two-way ANOVA showed *p* < 0.0001 for *BCL2*; *p* < 0.0001 for *BAX*, and *p* = 0.006 for *BAK1*. In **a**–c, each dot represents result from an individual patient and data are presented as the mean ± SEM; statistical significance shown was determined using two-tailed Student’s *T* tests (*p < 0.05; **p < 0.01; ***p < 0.001). Representative of two independent experiments with similar results are shown in **e**, **f**.

To validate the leukemogenic role of miR-126 observed in the murine inv(16) AML model, we knocked down miR-126 in human inv(16) AML CD34^+^ and CD34^−^ cells using shmiR-126 and assessed cell differentiation, proliferation, and apoptosis rates (Supplementary Fig. 7a). With a transduction efficiency of 55–70% (Supplementary Fig. 7b) and >70% miR-126 knockdown in GFP^+^-transduced cells (Fig. 5a), we detected a significantly increased frequency of more mature CD14^+^ and CD15^+^ cell subpopulation compared to control shRNA transduced cells (Supplementary Fig. 7c). In addition, we observed decreased CD34^+^ cell fractions in the G_0_ phase and increased in the G_1_ and S/G_2_/M phase (Fig. 5b and Supplementary Fig. 7d). Annexin V/DAPI staining showed increased spontaneous apoptosis rate after miR-126 knockdown (Fig. 5c, d). We verified that miR-126 knockdown resulted in upregulation of SPRED1, PLK2, decreased p-ERK, p-MYC (S62), and MYC levels in primary inv(16) AML CD34^+^ cells (Fig. 5e and Supplementary Fig. 7f–g). MYC plays a crucial role in regulating cell survival^58^ and repression of MYC is known to induce apoptosis in inv(16) AML cells^59^. In line with this, miR-126 knockdown resulted in significant downregulation of anti-apoptotic gene *BCL-2* and upregulation of pro-apoptotic genes *BAX*, *BAK* (Fig. 5e, g). Consequently, we observed increased cleavage of PARP and Caspase 3 (Fig. 5f) and DNA fragmentation (Supplementary Fig. 7h) in miR-126 knocked-down inv(16) AML CD34^+^ cells. Taken together, these results indicate that miR-126 knockdown resulted in a loss of quiescence, enhanced differentiation, and increased apoptosis of inv(16) AML CD34^+^ cells.

### Anti-miR-126 therapy inhibits leukemia progression and leukemia-initiating activity of LSCs

**Fig. 6:**
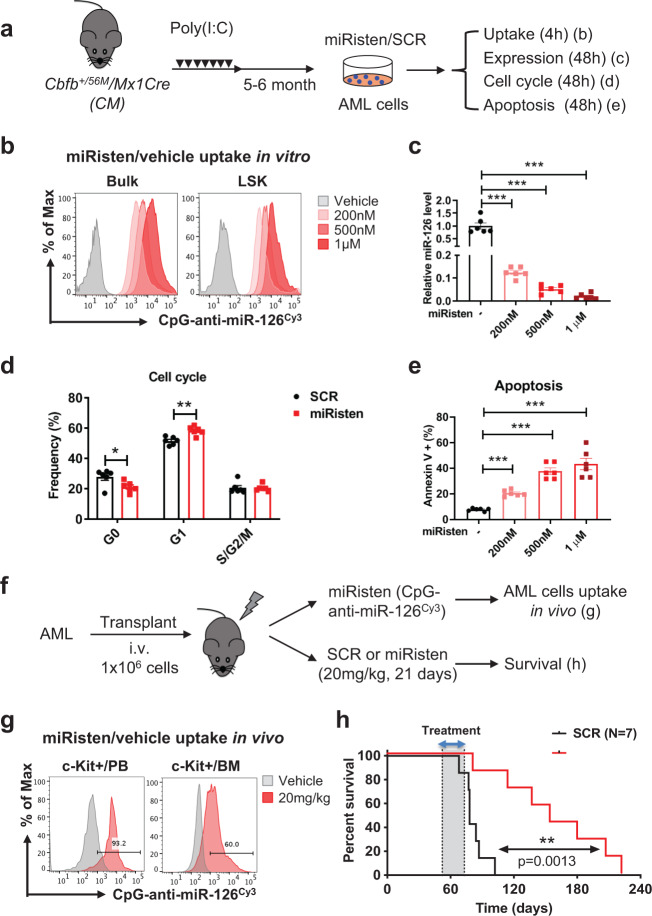
Targeting miR-126 by miRisten increases AML cell apoptosis and delays CM-AML progression. **a** Schematic of experimental design. AML cells isolated from CM induced leukemia mice were treated with miRisten or SCR control ex vivo and assessed for uptake, miR-126 expression, cell cycle, and apoptosis at various time points. **b** The frequency of uptake for miRisten (CpG-anti-miR-126^cy3^; red) or vehicle (gray) in bulk or LSK cells from CM leukemia BM. **c** Relative levels of miR-126 in CM AML cells after treatment with SCR control (black) or miRisten (200 nM, 500 nM, 1 μM; pink/red; *p* < 0.0001 for all doses) as determined by qPCR and normalized to SnoRNA234 expression. **d** Frequency (%) of G_0_, G_1_, or S/G_2_/M phases of cell cycle in CM AML cells after treatment with SCR control (black) or miRisten (red; G_0_ *p* = 0.031; G_1_ *p* = 0.0017). **e** Frequency (%) of apoptotic cells defined by Annexin V^+^ in CM AML cells after SCR (black) or miRisten (pink/red; *p* < 0.0001 for all doses) treatment. **f** Schematic of treatment regimen. CM AML cells (1 × 10^6^ cells/mouse) were transplanted into WT mice to generate a cohort of AML-bearing mice, which were randomly divided into two groups and treated with SCR control (20 mg/kg/dose, i.v., daily; *n* = 7) or miRisten (20 mg/kg/dose, i.v., daily; *n* = 7) for 21 days. **g** The frequency of uptake for miRisten (CpG-anti-miR-126^cy3^; red) or vehicle (gray) in c-Kit^+^ AML blasts in BM or PB 1 h after intravenous (i.v.) treatment with miRisten 20 mg/kg. **h** Kaplan–Meier survival curve of CM leukemic mice treated with SCR control (black line; *n* = 7; median survival 78 days) or miRisten (red line; *n* = 7; median survival 154 days). Dotted line with gray shade indicates treatment window; statistical difference for survival curves was determined using Log-rank (Mantel–Cox) test (*p* = 0.0013). Each dot in **c**, **d**, **e** represents result from an individual mouse and data are presented as the mean ± SEM; statistics were calculated using two-tailed Student’s *T* tests (*p < 0.05; **p < 0.01; ***p < 0.001).

Next, to test whether therapeutic targeting of aberrantly expressed miR-126 in CM-AML could have an anti-leukemic effect, we used a CpG-anti-miR-126 oligonucleotide inhibitor (miRisten) we previously designed^30^. First, we confirmed efficient uptake (>95%) in CM AML bulk and LSK cells (Fig. 6a, b) and effective downregulation (>90%) of miR-126 after miRisten treatment in vitro (500 nM and 1 μM) (Fig. 6c). We observed decreased G_0_ and increased G_1_ cell subfractions after miRisten treatment compared to scramble (SCR) control (Fig. 6d) and increased apoptosis rates in miRisten-treated compared to SCR-treated cells (Fig. 6e). Next, we confirmed in vivo uptake of Cy3-miRisten in AML blasts in PB (93.2%) and BM (60%) 1 h after intravenous (i.v.) treatment at 20 mg/kg (Fig. 6f, g). To evaluate the effects of miRisten on leukemia progression, we then generated a cohort of CM-AML mice (*n* = 14) that developed AML at a similar time by transplanting CM-AML cells into sublethally irradiated (4.5 Gy) WT recipients. When AML burden (as indicated by circulating cKit^+^ blasts) reached approximately 10% after 6 weeks, mice were randomized and treated either with miRisten or SCR control [20 mg/kg/dose/day, i.v.] for 21 days and monitored for survival (Fig. 6f). MiRisten-treated mice had a significantly longer survival compared with SCR-treated controls (Fig. 6h; *n* = 7 each group; median survival 154 vs. 78 days; *p* = 0.0013). Similar treatment with miRisten in normal WT mice did not cause any detectable changes in hematopoietic cell counts or frequency of lineage populations in the PB monitored over time (Supplementary Fig. 8), suggesting that miRisten have a selective anti-leukemia activity.

Similar to murine CM-AML cells, human inv(16) AML cells could efficiently uptake miRisten, effectively knocking down miR-126 and resulting in increased apoptosis (Supplementary Fig. 9a–e) and reduced quiescence of inv(16) AML CD34^+^ cells (Supplementary Fig. 9f, g). We confirmed that the effects of miRisten was indeed due to reduction of miR-126 since miRisten treatment in inv(16) AML CD34^+^ cells showed no additional effect when miR-126 was knocked down with shmiR-126 (Supplementary Fig. 9h, i). We also confirmed that miR-126 is highly expressed in t(8;21) AML CD34^+^ cells, and miRisten treatment similarly increased apoptosis and reduced quiescence of t(8;21) AML CD34^+^ cells (Supplementary Fig. 10).

**Fig. 7:**
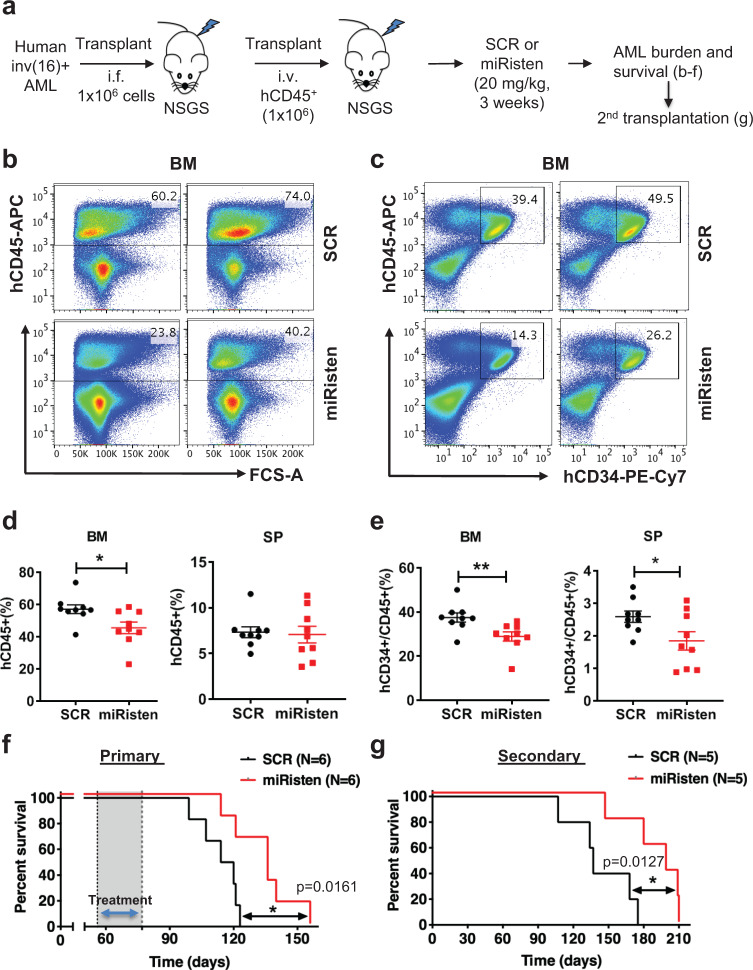
Administration of miRisten effectively inhibits AML propagation and leukemia-initiating activity in inv(16) AML PDX model. **a** Schematic of experimental design. Inv(16) AML PDX was established by directly injecting T cell-depleted primary AML cells (1 × 10^6^ cells/mouse) i.f. into irradiated NSGS and subsequently expanded into larger cohorts by secondary transplantation via i.v. injection. After 8 weeks, PDX mice were treated with SCR control (20 mg/kg/dose, i.v., daily) or miRisten (20 mg/kg/dose, i.v., daily) for 3 weeks and followed by assessment of human cell engraftment in BM and spleen. **b** Representative FACS plots showing gating strategy and frequency of hCD45^+^ cells in BM of mice treated with SCR control (top) or miRisten (bottom) for 3 weeks. **c** Representative FACS plots showing gating and frequency of hCD34^+^/CD45^+^ cells in BM of mice treated with SCR (top) or miRisten (bottom) for 3 weeks. **d** Frequency of hCD45^+^ AML cells in SCR (black) vs. miRisten (red) treated mice (*n* = 9/group) BM (*p* = 0.0222) or spleen (SP). **e** Frequency of hCD34^+^/CD45^+^ AML cells in SCR (black) vs. miRisten (red) treated mice (*n* = 9/group) BM (*p* = 0.01) or SP (*p* = 0.0385). **f** The Kaplan–Meier survival curve of mice treated with SCR (black line; *n* = 6; median survival 117 days) or miRisten (red line; *n* = 6; median survival 136 days; *p* = 0.0161) for 3 weeks starting at 8 weeks after transplant. Dotted line with gray shade indicates treatment window. **g** The Kaplan–Meier survival curve of second transplant recipients treated with SCR (black line; *n* = 5; median survival 137 days) or miRisten (red line; *n* = 5; median survival 199 days; *p* = 0.0127). The statistical significance for **f**, **g** was determined using log-rank (Mantel–Cox) test. Each dot in **d**, **e** represents result from an individual mouse and data are presented as the mean ± SEM; statistical significance was determined using two-tailed Student’s *T-*tests (*p < 0.05; **p < 0.01; ***p < 0.001).

We further evaluated the in vivo efficacy of miRisten in an inv(16) AML patient-derived xenograft (PDX) model. Primary inv(16) AML cells were expanded in NSGS mice by transplanting T cell-depleted AML blast samples (1 × 10^6^) into irradiated (2 Gy) mice via intrafemoral (i.f.) injection. A larger cohort of mice bearing human AML (*n* = 30) was then generated by transplanting human (h) CD45^+^ BM cells (1 × 10^6^) recovered from the above described PDXs into irradiated (2 Gy) NSGS recipients via intravenous injection (Fig. 7a). These secondary (s) PDXs were then treated with miRisten or SCR control (20 mg/kg/dose, i.v., daily) for 3 weeks. At the end of treatment, we observed significantly reduced hCD45^+^ burden in miRisten-treated BM compared to SCR control (Fig. 7a, b, d). Moreover, the CD34^+^/hCD45^+^ frequency was reduced (Fig. 7c, e) and the CD15^+^/hCD45^+^ cell frequency was increased in miRisten-treated sPDXs (Supplementary Fig. 11a–b), suggesting that miRisten treatment promoted partial differentiation of primitive inv(16) AML cells. MiRisten-treated sPDX mice (*n* = 6) also lived significantly longer than SCR-treated controls (Fig. 7f; *n* = 6; median survival 136 days vs. 117 days; *p* = 0.016). In secondary transplant experiments, the recipients (*n* = 5) of BM cells from miRsten-treated mice had a significantly longer survival than the recipients (*n* = 5) of BM cells from SCR-treated group (Fig. 7g; median survival 199 days vs. 137 days; *p* = 0.013). Altogether, these results indicate that in vivo administration of miRisten effectively inhibits AML propagation and impedes leukemia-initiating activity of inv(16) AML LSCs.

**Fig. 8:**
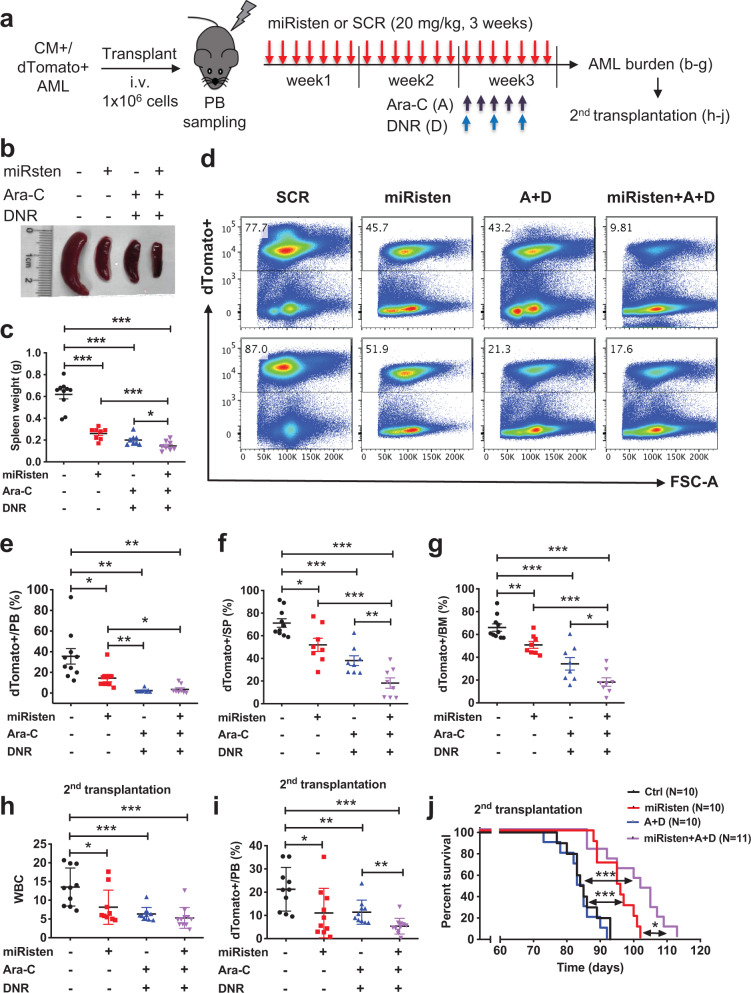
Anti-miR-126 therapy by miRisten effectively inhibits CM-AML burden and abrogates LSC activity. **a** Schematic of experimental design. *CM/tdTomato*^*+*^ AML cells (1 × 10^6^) were transplanted into cohorts of WTwild-type syngenic mice. After 8 weeks, mice were treated with SCR control or miRisten (20 mg/kg/dose, daily for 3 weeks), or a “5 + 3” chemotherapy regimen consisted of Ara-C (50 mg/kg/day, 5 days) plus DNR (1.5 mg/kg/day, 3 days), or miRisten and “5 + 3” chemotherapy combined. AML engraftment was analyzed 7 days after the last dose of treatment and BM cells were transplanted into second recipients, which were analyzed for engraftment at 7 weeks or monitored for leukemia onset and survival. **b** Representative images of spleens from recipients of SCR control (Ctrl), miRisten, A + D, or miRisten/A + D group. **c** Spleen weight from treated recipients of SCR Ctrl (black; *n* = 10), miRisten (red; *n* = 8; miRisten vs. Ctrl *p* < 0.0001), A + D (blue; *n* = 8; A + D vs. Ctrl *p* < 0.0001), or miRisten/A + D (purple; *n* = 8; miRisten/A + D vs. Ctrl *p* < 0.0001; miRisten/A + D vs. miRisten *p* = 0.0004; miRisten/A + D vs. A + D *p* = 0.0337). **d** Representative FACS plots showing gating strategy and frequency of dTomato^+^ AML cells in the BM of SCR control, miRisten, A + D, or miRisten/A + D group. **e** Frequency of dTomato^+^ AML cells in the PB of mice treated with SCR control (black; *n* = 10), miRisten (red; *n* = 8; miRisten vs. Ctrl *p* = 0.0324), A + D (blue; *n* = 8; A + D vs. Ctrl *p* = 0.0012; A + D vs. miRisten *p* = 0.0047), or miRisten/A + D (purple; *n* = 8; miRisten/A + D vs. Ctrl *p* = 0.0018; miRisten/A + D vs. miRisten *p* = 0.014). **f** Frequency of dTomato^+^ AML cells in the SP of mice treated with SCR control (black; *n* = 10), miRisten (red; *n* = 8; miRisten vs. Ctrl *p* = 0.0103), A + D (blue; *n* = 8; A + D vs. Ctrl *p* < 0.0001), or miRisten/A + D (purple; *n* = 8; miRisten/A + D vs. Ctrl *p* < 0.0001; miRisten/A + D vs. miRisten *p* = 0.0004; miRisten/A + D vs. A + D *p* = 0.0071). **g** Frequency of dTomato^+^ AML cells in the BM of mice treated with SCR control (black; *n* = 10), miRisten (red; *n* = 8; miRisten vs. Ctrl *p* = 0.0038), A + D (blue; *n* = 8; A + D vs. Ctrl *p* < 0.0001), or miRisten/A + D (purple; *n* = 8; miRisten/A + D vs. Ctrl *p* < 0.0001; miRisten/A + D vs. miRisten *p* < 0.0001; miRisten/A + D vs. A + D *p* = 0.0291). **h** White blood cell (WBC) counts of second transplant recipients of SCR control (black; *n* = 10), miRisten (red; *n* = 10; miRisten vs. Ctrl *p* = 0.0225), A + D (blue; *n* = 10; A + D vs. Ctrl *p* = 0.0005), or miRisten/A + D (purple; *n* = 11; miRisten/A + D vs. Ctrl *p* = 0.0002) treated mice at 7 weeks. **i** Frequency of dTomato^+^ AML cells in the PB of second transplant recipients treated with SCR Ctrl (black; *n* = 10), miRisten (red; *n* = 10; miRisten vs. Ctrl *p* = 0.0358), A + D (blue; *n* = 10; A + D vs. Ctrl *p* = 0.0098), or miRisten/A + D (purple; *n* = 11; miRisten/A + D vs. Ctrl *p* < 0.0001; miRisten/A + D vs. A + D *p* = 0.0049) at 7 weeks. **j** The Kaplan–Meier survival curve of second transplant recipients treated with SCR Ctrl (black line; *n* = 10; median survival 84.5 days), miRisten (red line; *n* = 10; median survival 95.5 days; miRisten vs. Ctrl *p* = 0.0004), A + D (blue line; *n* = 10; median survival 84 days), or miRisten/A + D (purple line; *n* = 11; median survival 102 days; miRisten/A + D vs. Ctrl *p* = 0.0002; miRisten/A + D vs. miRisten *p* = 0.0386). The statistical significance was determined using log-rank (Mantel–Cox) test. Each dot in **c**, **e**, **f**, **g**, **h**, **i** represents data from an individual mouse and presented as the mean ± SEM; statistics for all comparisons shown were determined using two-tailed *T-*tests (*p < 0.05; **p < 0.01; ***p < 0.001).

Given that chemotherapy remains the standard therapy for AML, we then tested the activity of miRisten combined with a chemotherapy “5 + 3” regimen using Ara-C (50 mg/kg/day, 5 days) and DNR (1.5 mg/kg/day, 3 days) in a CM AML mouse model with a tdTomato^+^ Cre-reporter (*Cbfb*^*56M/+*^/*Mx1-Cre/tdTomato*^*+*^*)*, which developed transplantable AML 3–6 months after poly(I:C) induction. AML cells from *Cbfb*^*56M/+*^/*Mx1-Cre/tdTomato*^*+*^ mice were transplanted into sublethally irradiated syngenic C57/B6 recipients (1 × 10^6^ cells), which were then treated with SCR control (20 mg/kg/dose, i.v., daily for 3 weeks; *n* = 10), miRisten (20 mg/kg/dose, i.v., daily for 3 weeks; *n* = 8), Ara-C + DNR (A + D; “5 + 3”; *n* = 8), or miRisten and A + D (during week 3; *n* = 8) combined (Fig. 8a). All treated mice were sacrificed 7 days after the final treatment administration and evaluated for AML burden in PB, BM, and spleen. MiRisten treatment significantly reduced splenic disease burden (Fig. 8b, c) as well as the frequency of dTomato^+^ AML cells in PB, spleen, and BM compared with SCR-treated controls (Fig. 8d–g). Combination of miRisten with A + D further decreased disease burden compared to miRisten alone (Fig. 8b–g). The frequency and cell number of BM myeloid progenitor (MP) subpopulations were significantly reduced by miRisten/A + D combined treatment compared to control or single (miRisten or A + D) treatment (Supplementary Fig. 12). To evaluate LSC activity, we transplanted BM cells from each treated group into secondary recipients (1 × 10^6^ cells; *n* = 10–11) and monitored for leukemia onset and survival. White blood cell count (WBC) and flow cytometry performed 7 weeks later showed significantly lower PB AML cell expansion in recipients of miRisten-treated cells compared to the SCR group (Fig. 8h, i). Further reduction was seen in the miRisten/A + D combined group compared to miRisten or A + D groups. Notably, transplants of miRisten-treated group showed significantly prolonged survival compared to the SCR-treated group (median survival 95.5 vs. 84.5 days; *p* = 0.0004) while the A + D-treated group showed no improvement of survival (Fig. 8j). Further prolonged survival was seen in transplants of combined miRisten/A + D treatment compared to the miRisten group (median survival 102 vs. 95.5 days; *p* = 0.0386). Collectively, these results indicate that systemic miRisten treatment decreased LSC activity in vivo and combination of miRisten with chemotherapy significantly inhibited AML progression and enhanced survival.

## Discussion

We demonstrate here a critical function of miR-126 in initiating and maintaining inv(16) AML by showing that genetic deletion of miR-126 prevented leukemia development in 50% of CM knock-in mice and significantly prolonged their survival. To develop an effective anti-miR-126 therapy, we employed the CpG-anti-miR126 inhibitor, miRisten^30^, which efficiently depleted miR-126 and significantly reduced AML blasts and leukemia-initiating LSCs both in inv(16) AML murine and PDX models.

High miR-126 was among the most significant miRNA expression signatures of CBF AML, which includes AML with t(8;21) and inv(16)^25,26^. A previous report by Li et al.^60^ showed that higher miR-126 is associated with poor prognosis in AML patients with t(8;21). However, the functional impact and mechanism of miR-126 dysregulation in inv(16) AML has been unclear. It has been suggested that hypomethylation of CpG islands in genomic regions hosting miR-126 is associated with increased expression of miR-126 in CBF leukemias^60^. Our studies reveal that miR-126, located within intron 7 of the *Egfl7* gene, is upregulated by the CM fusion protein via at least two distinct mechanisms. First, CM upregulates transcription of *Egfl7* and miR-126 through increased expression of GATA2, which functions to transactivate *EGFL7/miR-126*^39,40^). CBF transcription factors are known to cooperate with a variety of hematopoietic regulators, including GATA2, in transcriptional regulation^61^. In addition, Zhen et al.^62^ recently demonstrated that CM mainly act as a coactivator of RUNX1 target genes during leukemia development. Consistent with this, our results indicate that CM works together with RUNX1 and GATA2 to activate *Egfl7/miR-126* transcription. Consistent with a cooperative upregulation of *Egfl7*/miR-126, knockdown of *CM* or *Gata2* resulted in significant decrease of *Egfl7*/miR-126 levels. Furthermore, consistent with reduced leukemogenesis upon miR-126 deletion, *Gata2* deficiency also significantly delayed CM leukemogenesis^38^. Of note, transcriptional dysregulation of the *Egfl7/miR-126* promoter is not the only mechanism through which CM increases the level of miR-126. In fact, we demonstrated that CM enhances the biogenesis (i.e., maturation) of miR-126 through a previously unrecognized mechanism involving the HDAC8/RAN–XPO5 axis. We previously reported that *HDAC8* is upregulated in inv(16) AML and that CM interacts with HDAC8 and enhances HDAC8-mediated deacetylation of p53^45^. We show here that CM-HDAC8 interaction also enhances the stability of HDAC8 by preventing the binding of HDAC8 to EBP1, which otherwise mediates MDM2-depedent protein ubiquitination and degradation. We previously reported that *pre-miR-126* is exported from the nucleus to the cytoplasm through a “gatekeeper” RAN–XPO5 complex during miR-126 biogenesis^30^. Here, we discovered that HDAC8 deacetylates and enhances the activity of RAN protein, as supported by the increased binding of RAN to RCC1 and XPO5. This results in increased activity of the “gatekeeper” RAN–XPO5 complex with higher levels of *pre-miR-126* transported to the cytoplasm for completion of the last step of miR-126 biogenesis. Although t(8;21) AML also expresses high levels of miR-126 (Supplementary Fig. 10a), *EGFL7*, *GATA2*, and *HDAC8* levels were not significantly increased (Supplementary Fig. 10f), suggesting that miR-126 upregulation likely occurs via alternative mechanism(s). This and our recent reports highlight how distinct mechanisms of miR-126 dysregulation driven by specific leukemogenic mutations (i.e., *CBFB-MYH11*, *BCR-ABL*, *FLT3-ITD*) play a relevant role in leukemia pathobiology^30,31^. Furthermore, the HDAC8/RAN–XPO5 axis we discovered here can potentially contribute to miR-126 dysregulation in other subsets of leukemia or other cancer types with aberrant HDAC8 activity.

We and others have reported that high expression of miR-126 is an LSC-associated signature and a determinant of LSC stemness in acute and chronic myeloid leukemias^29,30^. By promoting cell cycle quiescence, miR-126 supports the maintenance and drug resistance of LSCs. On the other hand, forced expression of miR-126 in HSPC led to oncogenic transformation with features of B cell acute lymphoblastic leukemia^63^. In addition, Li et al. reported that overexpression and knockout of miR-126 both promote leukemogenesis induced by t(8;21)^60^. Our genetic studies revealed that aberrantly expressed miR-126 plays a critical role in initiating leukemogenesis and maintaining AML induced by inv(16) generated CM fusion protein. We and others have shown that CM expression impairs multi-lineage differentiation and induces expansion of preleukemic stem cells and progenitor cells; however, additional mutations are required for malignant transformation^32,50,64–66^. These aberrantly expanded CM preleukemic HSPCs display enhanced survival and serve as cellular reservoirs susceptible to acquisition of additional alternations needed for further clonal evolution^33^. Deletion of miR-126 significantly increases apoptosis especially in the LSK and Pre-Meg/E immunophenotypic subsets (Supplementary Fig. 3), which we previously reported are the leukemia-initiating populations prone to malignant transformation in CM knock-in mice^33^. MiR-126 deletion in CM LSK alters expression of multiple pathways important for malignant transformation or AML maintenance, highlighting the multifaceted impact of miR-126. Of note, we show that miR-126 functions to enhance MYC protein stability and activity via downregulating SPRED1 and PLK2, negative regulators of RAS–MEK–ERK pathway which in turn regulates p-MYC (S62). Pulikkan et al.^59^ previously demonstrated that CM maintains MYC expression by relieving RUNX1-mediated repression and that MYC expression is critical for the survival of inv(16) leukemia-initiating cells and AML cells. Our results reveal an additional miR-126-mediated regulatory axis converging on MYC, a critical pathway for CM-driven AML. Given the functional divergence of miR-126, the effects of miR-126on regulation of genes and pathways may be highly dependent on the specific miR-126 level within specific cellular compartment and/or oncogenic context. Our results confirmed that deletion of miR-126 did not result in detectable difference in the immunophenotype or frequencies of normal HSPC subsets (Supplementary Fig. 3a–d). Notably, Lechman et al. reported that miR-126 inhibition resulted in LSC exhaustion while promoting normal HSC expansion^22,29^. This divergent self-renewal outcome underscores the opportunity to selectively target LSCs by therapeutically targeting miR-126.

Importantly, we developed a miR-126 inhibitor, miRisten, which was efficiently taken up and effectively reduced miR-126 expression in AML blasts and stem/progenitor cells. We show the on-target anti-leukemia and anti-LSC efficacy of miRisten in preclinical models of inv(16) AML. In addition, miRisten exhibited similar activities against t(8;21) AML stem/progenitor cells. Furthermore, we demonstrate that the combination of miRisten with chemotherapy offered improved efficacy in inhibiting leukemia progression and eliminating LSCs. Concordant with our findings, previous studies showed that inhibiting miR-126 increased the sensitivity to anti-proliferative drugs or standard chemotherapy in cytogenetically normal AML^29^ and t(8,21) AML^60^. This study highlights therapeutically targeting miR-126 as a therapeutic approach for AML patients with inv (16).

## Methods

### Human samples

All AML samples used (see Supplementary Table 3) were from patients who were treated at City of Hope and normal bone marrow or mobilized peripheral blood stem cells were obtained from healthy (HL) donors. Samples were acquired with signed informed consent and the procedure was approved by the City of Hope Institutional Review Board, in accordance with an assurance filed with and approved by the Department of Health and Human Services.

### Isolation of mononuclear cells from patient samples

The specimen was transferred to a 50 mL conical tube and the volume was brought up to 25 mL using warm Dulbecco’s phosphate-buffered saline phosphate-buffered saline (DPBS) with 2% fetal bovine serum (FBS). The specimen was layered on the top of 20 mL Ficoll-Paque Plus in a 50 mL conical tube. Then, the tube was centrifuged at 300*g* for 30 min without break. The layer containing mononuclear cells and plasma was carefully transferred to a 50 mL conical tube and the volume was brought up to 50 mL with warm DPBS. The tube was then centrifuge at 1341*g* for 8 min. After discard the supernatant, the pellet was resuspended in 10 mL of warm DPBS. The cell number and viability were assessed and CD34^+^ cells were enriched using StemSep™ Human CD34 Positive Selection reagents (Stemcell Technologies, 14756).

### Mice

*Cbfb*^*56M/+*^^32,33^ (available upon request), *miR126*^*flox/flox*^^34^ (provided by Dr. Calvin J. Kuo, Standford University), and *Mx1-Cre*^67^ (Jackson Laboratory, Stock No. 003556) mice were backcrossed to C57BL/6 for more than ten generations. Ai14 Cre tdTomato reporter mice (Stock No. 007914) and C57BL/6J mice (Stock No. 000664) were purchased from the Jackson Laboratory. To induce *miR-126* deletion and/or CM expression, *Cbfb*^*56M/+*^*/miR126*^*flox/flox*^*/Mx1-Cre* mice and *Cbfb*^*56M/+*^*/Mx1-Cre* (6–8-week-old, both females and males) were injected intraperitoneally (i.p.) with polyinosinic–polycytidylic acid [poly (I:C)] (InvivoGen, tlrl-picw-250) at 14 mg/kg/dose every other day for a total of seven doses. Age-matched *Cbfb*^*56M/+*^*, miR126*^*flox/flox*^, or *Cbfb*^*56M/+*^*/miR126*^*flox/flox*^ littermates were similarly poly (I:C) treated and used as controls. Preleukemic analysis was performed 6 weeks after the last dose of poly (I:C) injection. Mice were monitored for leukemia development and survival for up to 1 year. AML transplant was performed via tail vein i.v. injection into sublethally irradiated (X-RAD 320-Precision X-Ray; 4.5 Gy) 6–8 week-old syngenic C57BL/6 wild-type (WT) mice. In vivo administration of miRisten or scramble (SCR) control was performed by daily retro-orbital i.v. injection. Mice were maintained on 12 h light/12 h dark cycles under 18–23 °C ambient temperature with 40–60% humidity in an Association for Assessment and Accreditation of Laboratory Animal Care-accredited animal facility. All experimental procedures were performed in accordance with federal and state government guidelines and established institutional guidelines and protocols approved by the Institutional Animal Care and Use Committee at City of Hope.

### Cell isolation and flow cytometry

BM mononuclear cells were collected from femurs, tibias, and pelvis using a mortar and pestle^33,36,37^. For fluorescence activated cell-sorting (FACS) analyses, cells were stained with fluorescently labeled antibodies in phosphate-buffered saline (PBS) with 0.5% bovine serum albumin (BSA) for 15 min at 4 °C. Antibodies used included are listed in Supplementary Table 4. Flow cytometry was performed using a 5-laser, BD LSRFortessa™ X-20 cell analyzer. For cell sorting, lineage-negative (Lin^−^) cells were first enriched using EasySep™ negative selection reagents (Stemcell Technologies, 18000) and then sorted on a 5-Laser, BD FACSAria Fusion Cell Sorter. Acquired data were analyzed by Flowjo software 10.6.1. Phenotypic HSPC populations included long-term HSCs (LT-HSC; Lin^−^cKit^+^Sca1^+^CD48^−^CD150^+^), short-term HSCs (ST-HSC; Lin^−^cKit^+^Sca1^+^CD48^−^CD150^−^), multipotent progenitors (MPP; Lin^−^cKit^+^Sca1^+^CD48^+^CD150^+/−^), LSK (Lin^−^cKit^+^Sca1^+^), pre-granulocyte–macrophage (Pre-GM; Lin^−^ckit^+^Sca1^−^CD16/32^−/lo^CD105^−^CD150^−^), granulocyte–macrophage progenitors (GMP; Lin^−^ckit^+^Sca1^−^CD16/32^+^CD150^−^), pre-megakaryocyte/erythrocyte (Pre-Meg/E; Lin^−^ckit^+^Sca1^−^CD16/32^−/lo^CD105^−^CD150^+^), and erythroid progenitors (EP; Lin^−^ckit^+^Sca1^−^CD16/32^−/lo^CD105^+^, including Pre-CFU-E, CFU-E, Pro-erythrocytes)^33,36,37^.

### RNA isolation and reverse transcription

Total RNA, including miRNA, was isolated using AllPrep DNA/RNA/miRNA Universal Kit (Qiagen, 80224) following the manufacturer’s protocol. First-strand cDNA was generated using SuperScript™ IV Reverse Transcriptase (Thermo Fisher Scientific, 18090010). Reverse transcription was performed using Applied Biosystems® TaqMan® MicroRNA Reverse Transcription Kit (Thermo Fisher Scientific, 4366596) and the gene specific primers [miR-126 (002228), Sno234 (001234), and RNU44 (001094)] provided by TaqMan® MicroRNA Assay.

### Quantitative PCR

Quantitative PCR was performed using TaqMan™ Universal Master Mix II, with UNG (Thermo Fisher Scientific, 4440038) or SYBR™ Green PCR Master Mix (Thermo Fisher Scientific, 4368577). TaqMan Assays and primer sequences are listed in Supplementary Tables 5 and 6. Results were normalized to internal reference genes by the Ct method, and relative expression was calculated as 2^−ΔCt^.

### Design and synthesis of miRisten (CpG-anti-miR-126)

The partially phosphothioated anti-miR-126 or scrambled (SCR) RNA was linked using 5 U of C3 carbon chain linker, (CH_2_)_3_ (indicated by “x”). The constructs were also conjugated with Cy3 to track the internalization in cells by flow cytometry. The sequences were as follows: CpG-anti-miR-126: 5′-G*G*TGCATCGATGCAGG*G*G*G*GxxxxxmCmGmCmAmUmUmAmUmUmAmC mUmCmAmCmGmGmUmAmCmGmA-3′; CpG-SCR: 5′-G*G*TGCATCGATGCAGG*G*G*G*G xxxxxmGmUmAmGmAmAmCmCmGmUmAmCmUmCmGmUmCmAmCmUmUmA-3′, where the asterisk (*) indicates phosphorothioation. One nonbridging atom of oxygen on phosphate was replaced with sulfur. “m” indicates the 2′-*O*-methyl analog of the nucleotide.

### Xenograft mouse model

Human inv(16) AML cells were transplanted via i.f. injection into sublethally irradiated (2 Gy) 6–8-week-old NOD/SCID/*IL-2R-γ*^*−/−*^/Tg (CMV-IL3, CSF2, KITLG) mice (NSGS) purchased from the Jackson Laboratory (Stock No. 013062). Leukemia BM cells were collected and transplanted (1 × 10^6^ cells) via tail vein i.v. injection into a larger cohort of NSGS mice. Engraftment of human (h)CD45^+^ cells in PB was monitored by flow cytometry at 6–8 weeks after transplantation. Mice were treated with miRisten or SCR control by retro-orbital i.v. injection (20 mg/kg/dose) daily for 21 days. This dose and schedule were selected based on empirical testing showing effective knockdown, efficacy, tolerability, and feasibility of drug synthesis. After treatment, PB and BM cells were collected and assessed for AML burden by flow cytometry (see antibodies in Supplementary Table 4).

### Virus production and transduction of primary AML and 32D cells

The miRZip anti-miR-126-3p-GFP co-expressing plasmid (CS940MZ-1, a custom order from System Biosciences, with EEF1A1 promoter for anti-miR-126-3p) and control plasmid (MZIP000-PA-1, miRZip negative control) were purchased from System Biosciences. Virus were produced and transduced as previously described^30^. Briefly, primary AML or enriched CD34+ cells were cultured in Gibco™ IMDM (Thermo Fisher Scientific, 12440061) supplemented with 20% FBS and growth factors including interleukin 3 (IL-3, 25 ng/mL), interleukin 6 (IL-6, 10 ng/mL), Fms-like tyrosine kinase 3 ligand (Flt-3 ligand, 100 ng/mL), stem cell factor (SCF, 50 ng/mL), and thrombopoietin (100 ng/mL) overnight before transduction. Transduction was performed with a multiplicity of infection (MOI) of 25 by spinoculation in the presence of 1× TransDux MAX™ (System Biosciences, LV860A-1). 32D cells were cultured in RPMI-1640 supplemented with 10% FBS, 20% WEHI-3 conditioned media, and antibiotics at 37 °C with 5% CO_2_ and were transduced with MSCV-IRES-GFP (MIG)-based retroviruses or lentiviruses (CD530, pLKO.1 or HIV-7) (MOI = 10) by spinoculation in the presence of 8 ug/mL polybrene (American Bioanalytical, AB01643-00001). Two days after infection, GFP^+^ or RFP^+^ cells was sorted on a 5-Laser, BD FACSAria Fusion Cell Sorter. The target sequences for shRNAs are shown in Supplementary Table 7.

### Transfection of 32D cells with synthetic small interfering RNA (siRNA) oligonucleotides

32D cells were transfected by siEbp1 (the siGENOME SMARTpool, J-00860-05), which purchased from Thermo Fisher Scientific and scrambled control RNA (siCtrl) using electroporation with SF Cell Line 4D-Nucleofector^TM^ X Kit (Lonza, V4XC-2032) via program CV-137 following the manufacturer’s protocol. The target sequences for siRNAs are shown in Supplementary Table 7.

### Chromatin immunoprecipitation

Cells are fixed with formaldehyde and lysed according to the SimpleChIP^®^ Plus Enzymatic Chromatin IP Kit (Magnetic Beads, Cell Signaling Technology, 9005). After chromatin is digested, immunoprecipitations are performed using ChIP-validated antibodies: Anti-FLAG (Cell Signaling Technology, 14793), CBFb Antibody-ChIP-seq Grade (Diagenode, C15310002), Recombinant Anti-RUNX1/AML1 antibody (Abcam, ab272456), Anti-GATA2 antibody-ChIP Grade (Abcam, ab22849), and ChIP-Grade Protein G Magnetic Beads. After reversal of protein-DNA cross-links, the DNA is purified using DNA purification spin columns, following with quantitative real-time PCR. Fold enrichment was calculated with equation: 2^−(C[T] IP Sample – C[T] IP IgG)^.

### Immunocytochemistry

Cells were collected, washed in ice-cold PBS and mounted on glass slides using a Cytocentrifuge (CytoSpin4, 91*g*, 10 min). Cells were then washed with PBS, fixed in 4% paraformaldehyde for 15 min and permeabilized in 0.5% Triton X-100 for 15 min. Non-specific epitopes were blocked with 5% BSA for 30 min. Primary antibodies used are listed in Supplementary Table 8. Secondary anti-mouse/rabbit/goat-Alexa 594/488/647 antibodies were purchased from Thermo Fisher Scientific. Cell images were acquired using a Zeiss confocal laser-scanning-microscope (Zeiss LSM 800). Nuclei were counterstained with ProLong^TM^ Gold Antifade Mountant with DAPI (Thermo Fisher Scientific, P36931).

### Western blotting and immunoprecipitation

Cells were washed and harvested in ice-cold PBS and subsequently lysed in radioimmunoprecipitation assay (RIPA) buffer containing 10 mM protease inhibitor cocktail (Thermo Fisher Scientific, 78429). For immunoprecipitation, 500 µg to 1 mg of cell lysates were incubated with indicated antibody for overnight at 4 °C. Fifty microliters of Protein A/G agarose beads (Cell Signaling Technology, 9863; 37478) were added, and then incubated at 4 °C for 3 h. For immunoblotting, immunoprecipitated complex or 50 µg of each cell lysate was separated on NuPAGE 4–12% gradient gels (Thermo Fisher Scientific), and immunocomplexes were visualized with enhanced chemiluminescence reagent (Thermo Fisher Scientific, 34095). The information for antibodies used is presented in Supplementary Table 8.

### Ubiquitination assay

Cells were washed twice with PBS and then 50 μL of lysis buffer (Tris-buffered saline [TBS] and 2% sodium dodecyl sulfate [SDS]) was added and boiled. Two hundred and fifty microliters TBS supplemented with 1% Triton X‐100 was added and the mixture was sonicated. After centrifugation, the supernatant was immunoprecipitated using anti‐HDAC8 antibody following capture by protein G plus protein A agarose mixture (Calbiochem®, IP05). The bound proteins were eluted using 4× SDS sample buffer boiling at 100 °C for 5 min, resolved on NuPAGE 4–12% gradient gels (Thermo Fisher Scientific), and detected by anti-ubiquitin antibody.

### RAN activation assay

RAN activation assay (Cell Biolabs, STA-409) was performed to measure activities of RAN according to the manufacturer’s guidance. Briefly, the cells were washed twice with ice-cold PBS and lysed in lysis buffer with passing through a 27 1/2-gauge serine to shear the genomic DNA. The lysate was centrifuged, and supernatant was collected. RanBP1 PBD agarose bead was added and the complex was incubated at 4 °C for 1 h with gentle agitation. For positive and negative controls, GTPγS and GDP were added, respectively. Then the beads were collected, resuspended with 2× SDS-PAGE sample buffers, and boiled for 5 min. Twenty microliters of pulldown sample was loaded to NuPAGE 4–12% gradient gels (Thermo Fisher Scientific) and detected using anti-RAN antibody.

### Luciferase reporter assay

293 T cells were co-transfected with pGL3Basic-miR126-EGFL7-Promoter plasmid (Addgene, 32244) derived from pGL3.0 vector and Renilla luciferase reporter vector (phRL-TK) combined with pMIG-CM or pMIG-CBFb and pMIG-RUNX1 or empty control vector. After transfection (48 h), the activities of firefly and Renilla luciferases were measured sequentially from a single sample using the Dual-Luciferase® Reporter Assay System (Promega, E1910) according to the manufacturer’s protocol. Briefly, cultured cells were rinsed with 1× PBS and lysed by 1× Passive Lysis Buffer (PLB) solution (Promega, E1941). Homogeneous lysates were prepared by manually scraping the cells from culture wells in the presence of 1× PLB and then transferred into a tube. The firefly luciferase reporter is measured first by adding Luciferase Assay Reagent II (LAR II) to generate a stabilized luminescent signal. After quantifying the firefly luminescence, this reaction is quenched, and the Renilla luciferase reaction is simultaneously initiated by adding Stop & Glo® Reagent to the same tube and then measured Renilla luminescence. Measurements were read using a white, flat bottom 96-well plate (Costar, 3912) on a GloMax® 20/20 Luminometer. The normalized luciferase ratio is obtained for each well by calculating “firefly reporter activity/Renilla reporter activity”.

### Small RNA library preparation and next-generation sequencing (NGS)

All libraries were prepared using the Illumina TruSeq Small RNA protocol with minor modification following the manufacturer’s instructions. Briefly, 280 ng of pooled total RNA was ligated to the sRNA 3′ adaptor (TCT GGA ATT CTC GGG TGC CAA GGA ACT CC) with T4 RNA Ligase 2, truncated (New England BioLabs) for 1 h at 22 °C, and subsequently ligated to a 5′ adaptor (0.5 μL of 5 μM per reaction): GUUCAGAGUUCUACAGUCCGACGAUCNNN with T4 RNA ligase1 (New England BioLabs) for 1 h at 20 °C. The constructed smRNA library was first reverse-transcribed using GX1 (5′-GGAGTTCCTTGGCACCCGAGA) as the RT primer and then subjected to PCR amplification for 13 cycles, using primers GX1 (5′-CAAGCAGAAGACGGCATACGAGAT[NNNNNN]GTGACTGGAGTTCCTTGGCACCCGAGAATTCCA) and GX2 (5′-AATGATACGGCGACCACCGAGATCTACACTCTTTCCCTACACGACGCT CTTCCGATCT) then followed by 6% TBE PAGE gel purification with size selection (for targeted small RNAs of 17–35 nt). The purified library was quantified using qPCR with a forward primer (5′-CAAGCAGAAGACGGCATACG) and a reverse primer (5′-AATGATACGGCGACCACCGA). Individual libraries were prepared using a unique index primer in order to allow for pooling of multiple samples prior to sequencing. The library was quantified using qPCR. Sequencing of 50 cycles was performed on a HiSeq 2500 (Illumina Inc., San Diego, CA), and image processing and base calling were conducted using Illumina’s pipeline.

### miRNA-seq data analysis

Raw small RNA sequences were trimmed to remove the 3′-adapter (TCTGGAATTCTCGGGTGCCAAGGAACTCC) using cutadapt v0.9.3. Reads longer than 16 bp after trimming were aligned to mouse genome assembly mm9 using Bowtie v 0.12.7 with default settings. The expression level of mouse mature miRNAs from Sanger mirBase v18 were counted as previously described^68^. The counts of miRNAs were then normalized by the trimmed mean of M-values (TMM) method and counts per million (CPM) values were calculated by Bioconductor package “edgeR” v3.4.2.

### RNA-seq and bioinformatics

Total RNA was extracted using the AllPrep DNA/RNA/miRNA Universal Kit (Qiagen, 80224); quality and quantity were estimated using BioAnalyser Systems (Agilent Technologies). Samples with a RIN >8.0 were included. External RNA Controls Consortium (ERCC) Spike-In Control Mix (Thermo Fisher Scientific, 4456740) was added to all samples per the manufacturer’s recommendations, although these were not used for downstream analyses. Sequencing libraries were constructed using the KAPA RNA HyperPrep Kit with RiboErase (HMR) (Roche, KK8560), loaded on to a cBot system for cluster generation, and sequenced on a Hiseq 2500 System (Illumina) with single end 51-bp for mRNA-seq to a nominal depth of 50 million reads. Raw RNA-seq sequences were subjected to adapter trimming using Trimmomatic v0.38 and poly(A) tails were removed using FASTP v0.19.4. The trimmed reads were aligned to mouse genome mm10 using Tophat v2.0.8 with default settings. Expression level of RefSeq gene (downloaded on 02/13/2020) were counted using HTSeq count v0.6.1. The raw count data were normalized by the TMM method using Bioconductor package “edgeR” v3.20.9. Differential expression analysis was carried out using the quasi-likelihood (QL) *F*-test implemented in “edgeR” to determine the differentially expressed genes, with the cutoff of average RPKM in one group ≥1, *p* values ≤0.01 and fold change ≥1.5. The gene set enrichment analysis (GSEA^69^) was performed by ClusterProfiler v3.10.1 to identify the affected GO, hallmark and KEGG pathways from MSigDB v7^70^, using the pre-ranked gene list sorted by the −log10(*p* value) with a sign determined by the fold change direction.

### Statistics and reproducibility

Comparison between groups was performed by a two-tailed, paired or unpaired Student’s *T*-test, one-way or two-way ANOVA for normal distributions. The log-rank test was used to assess significant differences between survival curves. All statistical analyses were performed using Prism version 8.0 software (GraphPad Software). Sample sizes chosen are indicated in the individual figure legends and were not based on formal power calculations to detect prespecified effect sizes. A *p* value less than 0.05 was considered statistically significant. All experiments are repeated independently with similar results at least two times.

### Reporting summary

Further information on research design is available in the Nature Research Reporting Summary linked to this article.

## Supplementary information

Supplementary Information

Reporting Summary

## Data Availability

The raw data and processed RNA-seq data for LSK cells generated in this study have been deposited in the GEO repository under accession number GSE184015, which can be assessed with no restriction. The raw data and processed RNA-seq data associated with Fig. 1D, S1C was deposited under GEO accession number GSE133642, which can be assessed with no restriction. Raw miRNA-seq data associated with Fig. 1C were deposited to GEO under accession number GSE173785. The uncropped blots generated in this study are provided in the Supplementary Information/Source data file. Source data are provided with this paper.

## Source data

Source Data
