# Supplementary material for: Targeting miR-126 in inv(16) acute myeloid leukemia inhibits leukemia development and leukemia stem cell maintenance
Source: Nat Commun. 2021 Oct 22;12:6154. doi: 10.1038/s41467-021-26420-7 (PMC8536759; doi:10.1038/s41467-021-26420-7)
Supplement: Supplementary file 1 — Supplementary Information [file 41467_2021_26420_MOESM1_ESM.pdf]

Targeting miR-126 in inv(16) acute myeloid leukemia inhibits leukemia development and leukemia stem cell maintenance

Lianjun Zhang[1]†, Le Xuan Truong Nguyen[1]†, Ying-Chieh Chen[1], Dijiong Wu[2], Guerry J Cook[1], Dinh Hoa Hoang[1], Casey J Brewer[1], Xin He[1], Haojie Dong[1], Shu Li[3], Man Li[1], Dandan Zhao[1], Jing Qi[1], Wei-Kai Hua[1], Qi Cai[1], Emily Carnahan[1], Wei Chen[4], Xiwei Wu[4], Piotr Swiderski[5], Russell C Rockne[6], Marcin Kortylewski[7], Ling Li[1], Bin Zhang[1], Guido Marcucci[1] and Ya-Huei Kuo[1]*

Supplementary information includes the following:

Supplementary Figures 1-12

Supplementary Table 1. Differentially expressed genes in CM/miR-126$^{\Delta/\Delta}$ LSK versus CM LSK

Supplementary Table 2. Top 13 most differentially enriched pathways in CM/miR-126$^{\Delta/\Delta}$ LSK versus CM LSK

Supplementary Table 3. Characteristics of patient samples used in this study

Supplementary Table 4. Information for antibodies used for flow cytometry

Supplementary Table 5. TaqMan gene expression assays used for qPCR analysis

Supplementary Table 6. Primer sequences used for qPCR analysis

Supplementary Table 7. The sequences for siRNAs and shRNAs

Supplementary Table 8. List of antibodies used for IP, IB and CHIP analysis

Supplementary Figure 1

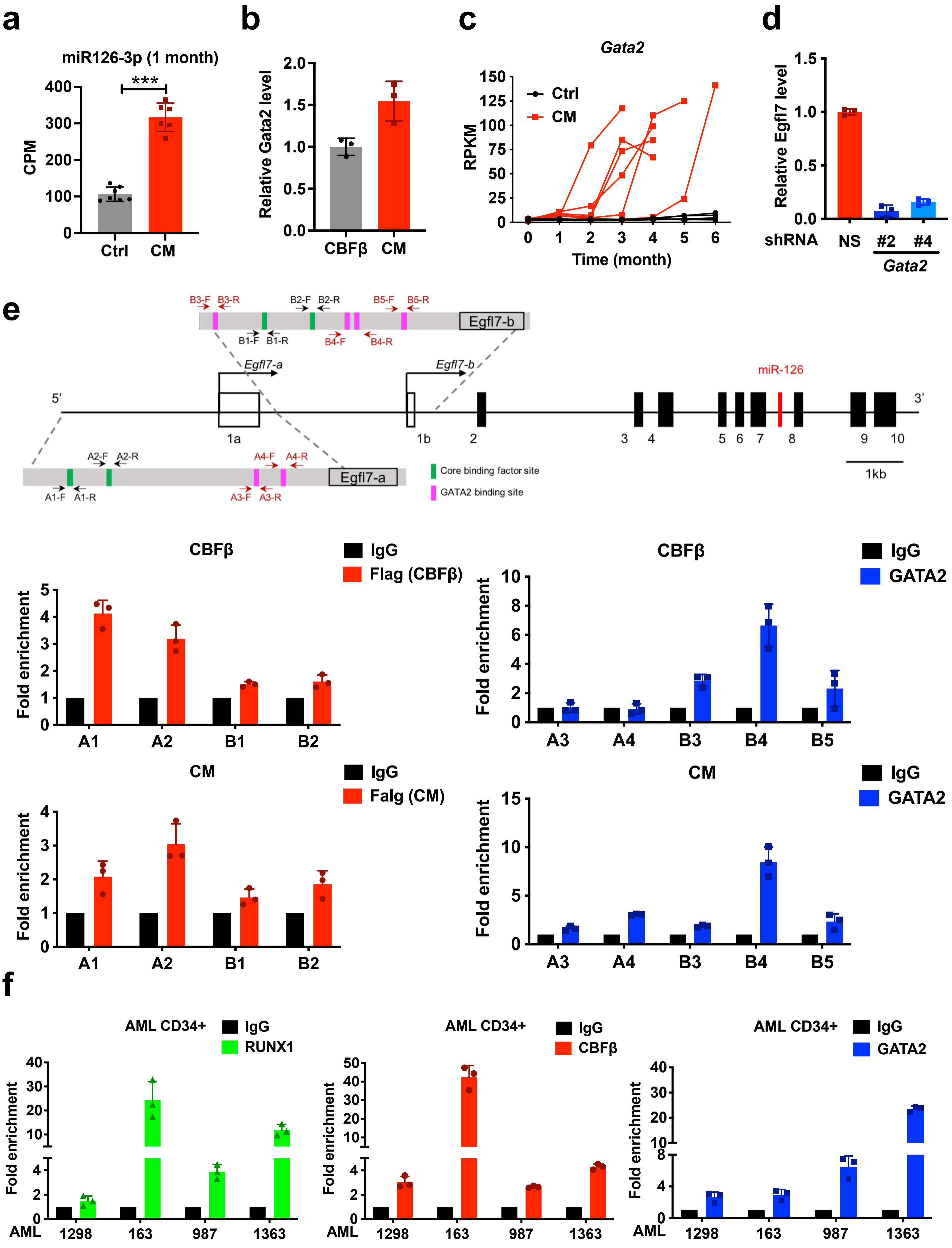

Supplementary Figure 1. CM up-regulates *Egfl7/miR-126* transcription in concert with *Gata2*.

(a) miR-126-3p counts per million (CPM) in PB of control (Ctrl; Black dots/gray bar; n=7) and CM (red dots/bar; n=6; p<0.0001) mice 1 month after induction.

(b) Relative *Gata2* levels in 32D-CBFβ (black) vs. 32D-CM (red) cells assessed by qPCR and normalized to *B2m*.

(c) Normalized reads per kilobase per million of transcript (RPKM) for *Gata2* in PB in control (black; n=7) and CM (red; n=6) mice over time until moribund with leukemia. Each line represents trajectory of one mouse; two-way ANOVA analysis showed Ctrl vs. CM p<0.0001.

(d) Relative level of *Egfl7* in leukemia BM cells transduced with NS control (red) or *Gata2* shRNA #2 (dark blue), *Gata2* shRNA #4 (light blue) as assessed by qPCR and normalized to *B2m.*

(e) Schematic diagram of predicted CBF (green) and GATA2 (magenta) binding sites in the *Egfl7* promoter region (top); and ChIP using anti-Flag (red) and anti-GATA2 (blue) antibody followed by qPCR using primers flanking four putative CBF binding sites (A1, A2, B1, B2) and six putative GATA2 binding sites (A3, A4, B3, B4, B5) in 32D-CBFβ and 32D-CM cells.

(f) ChIP using anti-RUNX1 (green), anti-CBFβ (red) and anti-GATA2 (blue) antibody followed by qPCR using primers flanking the reported RUNX1 binding site or the GATA2 binding site in inv(16) AML $CD34^+$ cells.

Data in (a), (b), (d), (e), (f) are presented as mean ± SD; shown are representative data of at least two independent experiments with 3 replicates. Each line in (c) represents the trajectory of an individual mouse.

Supplementary Figure 2

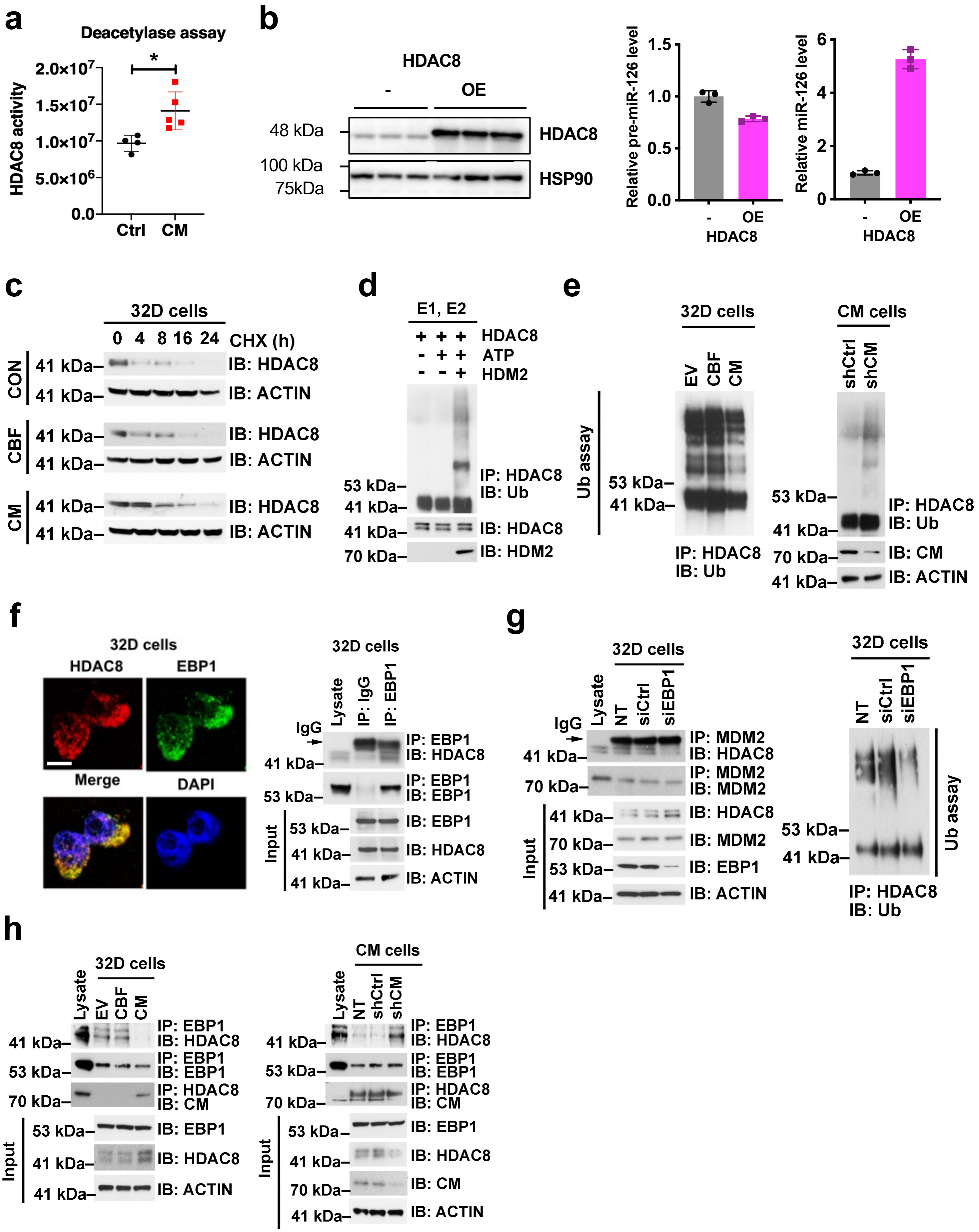

Supplementary Figure 2. CM enhances HDAC8 stability by preventing MDM2 mediated degradation.

(a) Deacetylase activity of pulled-down HDAC8 in BM samples of control (Ctrl; black; n=4) and CM leukemic mice (red; n=5; p=0.0157).

(b) Western blot analysis of HDAC8 in 32D cells with HDAC8 OE (left); relative levels of pre-miR-126, mature miR-126 in 32D cells with empty vector (black) or HDAC8 OE (pink) assessed by qPCR and normalized to *B2m*, snoRNA234, respectively (right).

(c) Time course IB with anti-HDAC8 or anti-Actin in 32D control cells (CON), *CBFb* or *CM* stably transduced cells treated with cycloheximide (CHX).

(d) *In vitro* ubiquitylation assay performed using ubiquitylation kit with HDAC8 and HDM2 recombinant protein (top). Loading control for HDAC8 and HDM2 expression is shown (bottom).

(e) HDAC8 ubiquitylation assay performed by IP with anti-HDAC8 and IB with anti-Ubiquitin antibodies using lysates from 32D cells, *CBFb* or *CM* stably transfected cells (left) or 32D-CM cells transduced with shCtrl or shCM lentivirus for 24 hours. WB of CM and β-Actin is shown on the bottom.

(f) Confocal microscopy showing IF co-staining with anti-HDAC8 (red) and anti-EBP1 (green) in 32D cells (left; scale bar 10 μm). IP with anti-IgG control or anti-EBP1 and IB with anti-HDAC8 in 32D cells.

(g) IP with anti-MDM2 and IB with anti-HDAC8 or IP with anti-HDAC8 and IB with anti-Ubiquitin antibodies in 32D cells transfected with siCtrl control or siEBP1 (20 nM) for 24h.

(h) IP with anti-EBP1 and IB with anti-HDAC8 antibodies (first and second panels) or IP with anti-HDAC8 and IB with anti-CM antibodies (third panel) in 32D parental cells, *CBFb* or *CM* stably transduced cells (lef). IP and IB with indicated antibodies in 32D-CM cells were transduced with shCtrl or shCM lentivirus for 24 hours. WB of HDAC8 and CM is shown on the bottom.

Each dot in (a) shows result from one mouse and the statistical significance was determined using two-tailed student's T tests (*p<0.05). Data are presented as mean ± SD in (b). Representative results from at least two experiments are shown in (b), (c), (d), (e), (f), (g), (h).

Supplementary Figure 3

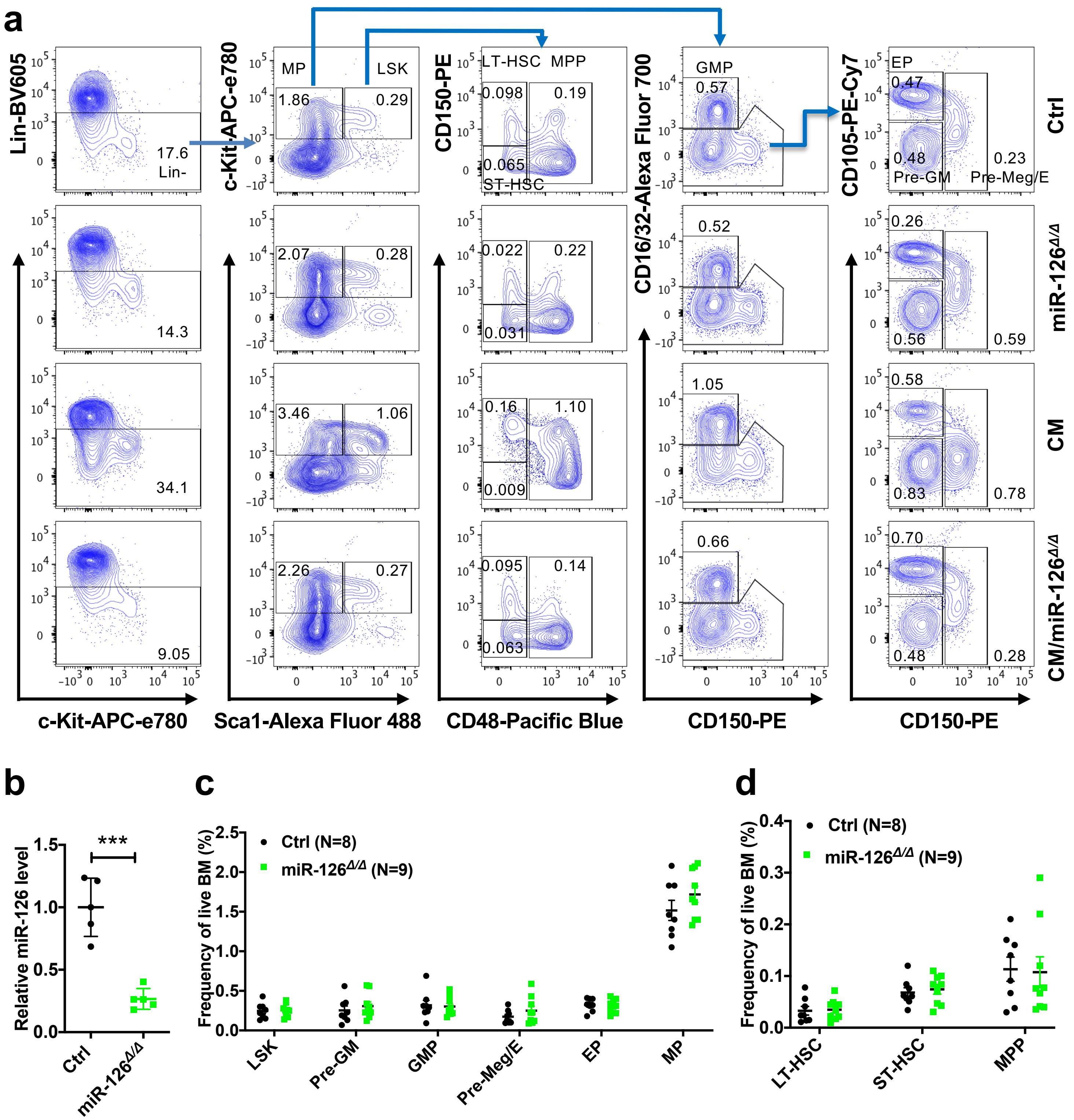

Supplementary Figure 3. MiR-126 contributes to CM induced expansion of preleukemic HSPC.

(a) Representative FACS plots showing gating strategy and frequency of phenotypic populations including LSK, LT-HSC, ST-HSC, MPP, GMP, pre-GM, pre-Meg/E, and EP in control (Ctrl), *miR-126* $^{\Delta/\Delta}$, *CM* and *CM/miR-126* $^{\Delta/\Delta}$ preleukemic BM. Similar gating strategy is used for all flow cytometry analysis and sorting of HSPC populations shown in Figure 1E, 3C-D, S4C.

(b) Relative expression levels of miR-126 in control (black; n=8) and *miR-126*$^{\Delta/\Delta}$ (green; n=9) BM cells, as assessed by qPCR and normalized to levels of SnoRNA234. Data are presented as the mean ± SD.

(c) Frequency of LSK, myeloid/erythroid progenitor populations in control (black; n=8) or *miR-126* $^{\Delta/\Delta}$ (green; n=9) BM.

(d) Frequency of LT-HSC, ST-HSC and MPP subsets in control (black; n=8) or *miR-126*$^{\Delta/\Delta}$ (green; n=9) BM.

Data are presented as the mean ± SEM. Each dot shows result from one mouse; statistical significance was assessed using two-tailed student's T test (***p< 0.001).

Supplementary Figure 4

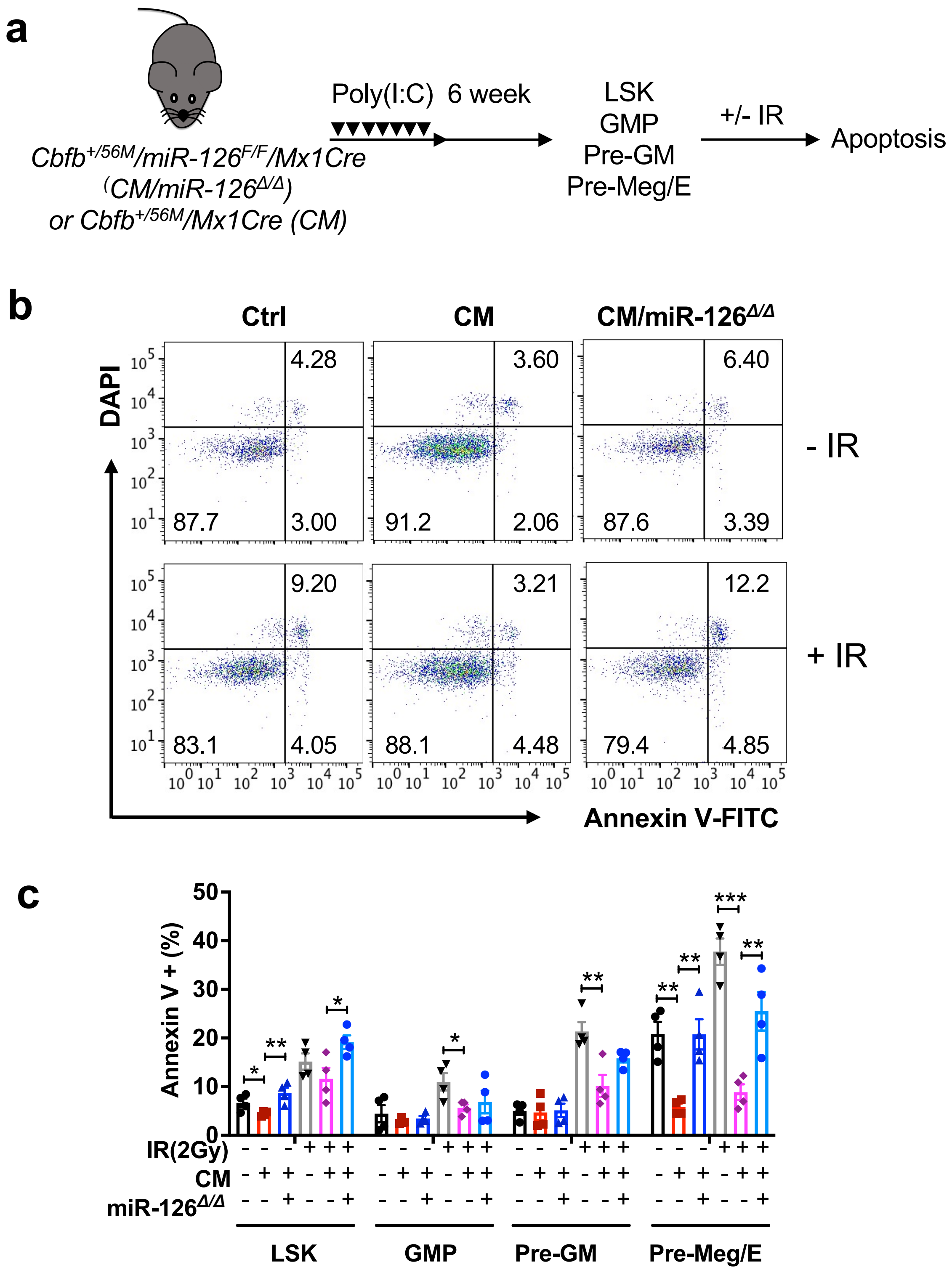

Supplementary Figure 4. MiR-126 contributes to survival of leukemia-initiating populations.

(a) Schematics of experimental strategy.

(b) Representative FACS plots showing gating strategy and frequency of Annexin V/DAPI staining in LSK.

(c) The frequency (%) of apoptotic cells defined by Annexin V^+ with or without IR (2 Gy; n=4 for each group) in control (black, gray), *CM* (red, magenta) and *CM/miR-126*$^{\Delta/\Delta}$ (dark blue, light blue) phenotypic HSPC subsets, including LSK (*CM* vs. Ctrl (-)IR p=0.04; *CM/miR-126*$^{\Delta/\Delta}$ vs. *CM* (-)IR p=0.007; *CM/miR-126*$^{\Delta/\Delta}$ vs. *CM* (+)IR p=0.03); GMP (*CM* vs. Ctrl (+)IR p=0.03); Pre-GM (*CM* vs. Ctrl (+)IR p=0.009); Pre-Meg/E (*CM* vs. Ctrl (-)IR p=0.001; *CM/miR-126*$^{\Delta/\Delta}$ vs. *CM* (-)IR p=0.003; *CM* vs. Ctrl (+)IR p<0.0001; *CM/miR-126*$^{\Delta/\Delta}$ vs. *CM* (+)IR p=0.008).

Data are presented as the mean ± SEM; statistical significance of all comparisons were determined using two-tailed T test (*p<0.05; **p<0.01; ***p<0.001).

Supplementary Figure 5

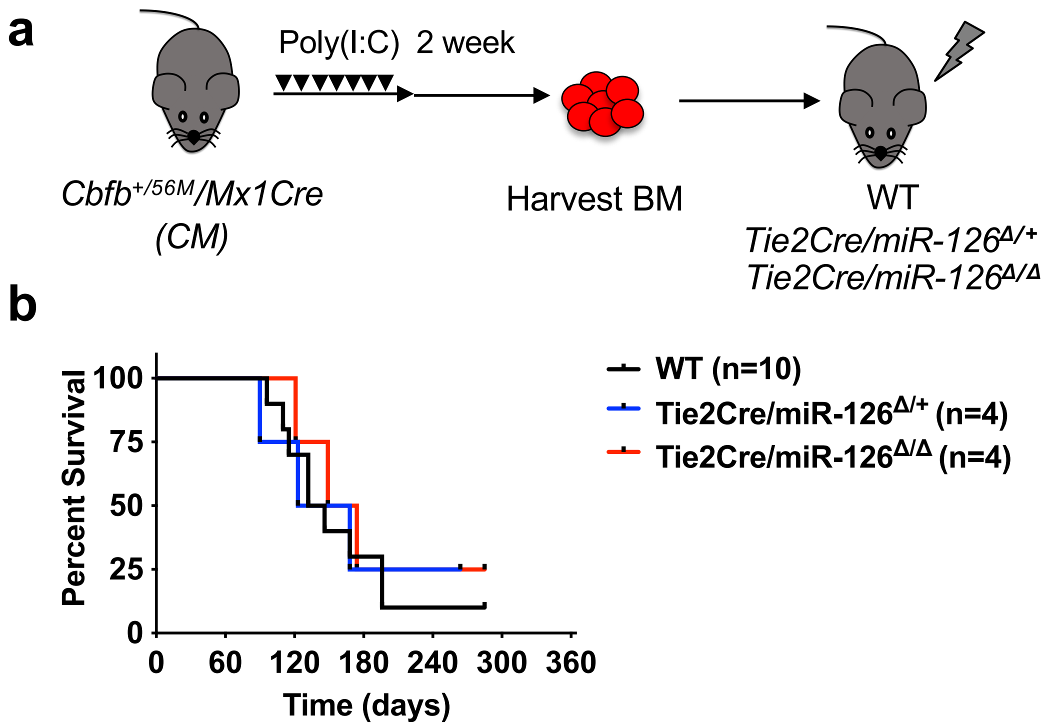

Supplementary Figure 5. Deletion of miR-126 in endothelial cells did not impact CM-driven leukemogenesis.
(a) Schematic of experimental design. CM-expressing BM HSPC was isolated from *Cbfb*$^{+/56M}$*/Mx1Cre* mice 2 weeks after 7 doses of poly(I:C) injection and transplanted into wild type (WT; n=10), *Tie2Cre/miR-126*$^{\Delta/+}$ (n=4), or *Tie2Cre/miR-126*$^{\Delta/\Delta}$ (n=4) recipients.
(b) Kaplan-Meier survival curve of recipient mice from each WT (n=10; black line), *Tie2Cre/miR-126*$^{\Delta/+}$ (n=4; blue line), or *Tie2Cre/miR-126*$^{\Delta/\Delta}$ (n=4; red line) monitored up to one year.

Supplementary Figure 6

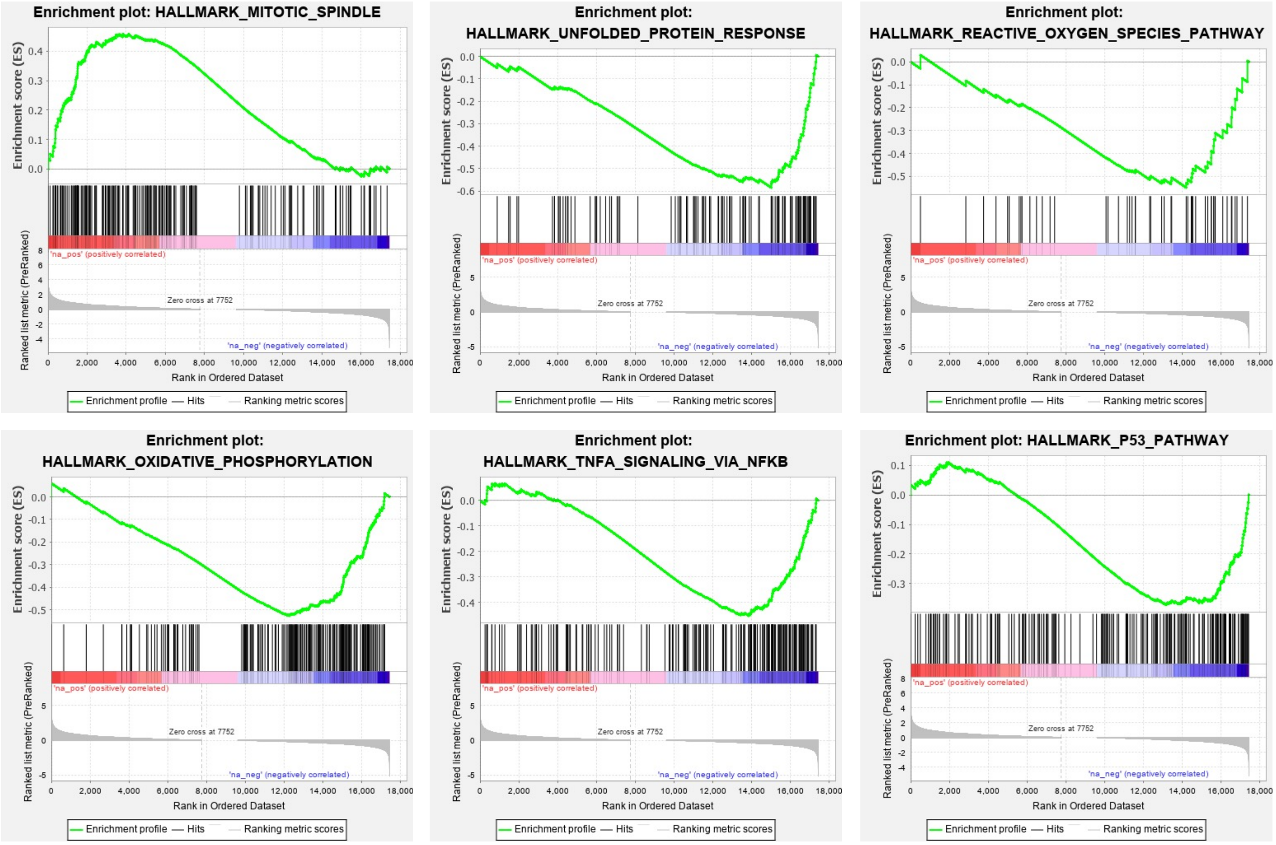

Supplementary Figure 6. Enrichment plots for most significantly enriched Hallmark Signature pathways in *CM/miR-126*$^{\Delta/\Delta}$ vs. *CM* LSK identified by GSEA of RNA-seq data.

Supplementary Figure 7

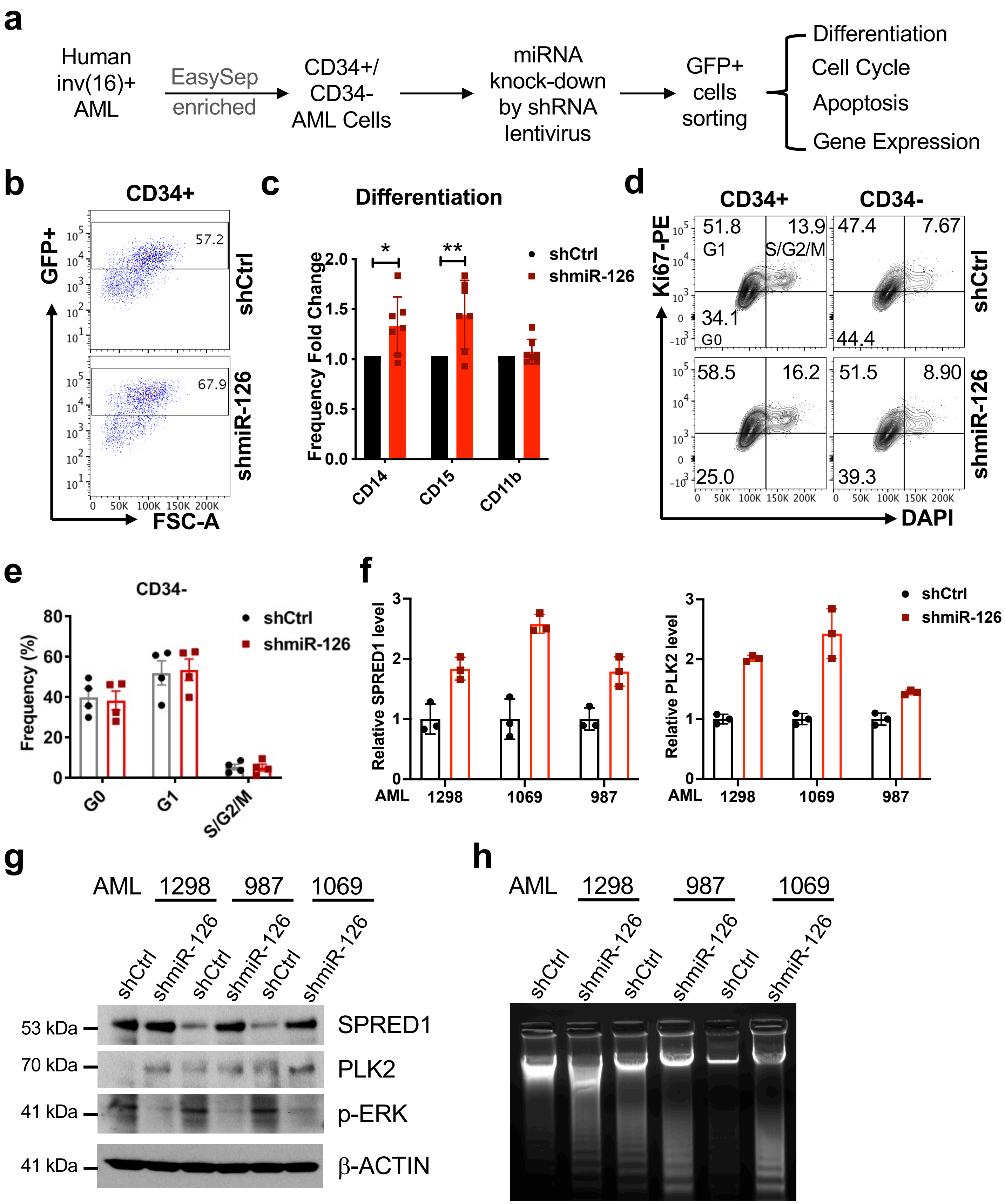

Supplementary Figure 7. Knockdown of miR-126 promotes apoptosis and reduces quiescence of primitive inv(16) AML cells.

(a) Schematic of experimental design. $CD34^+$ and $CD34^-$ cells were isolated from inv(16) AML patients, transduced with lentiviral vector (GFP^+) expressing shmiR-126 and sorted for assessment of differentiation, cell cycle and apoptosis.

(b) Representative FACS plots showing gating strategy and frequency of GFP^+ cells after transducing with shRNA control (shCtrl) or shmiR-126 in inv(16) AML $CD34^+$ cells.

(c) Fold change of the frequency of $CD14^+$ (p=0.011), $CD15^+$ (p=0.005) and $CD11b^+$ fractions detected 8 days after shCtrl (black) or shmiR-126 (red; n=7) transduction of inv(16) AML $CD34^+$ cells.

(d) Representative FACS plots showing gating strategy and frequency of Ki67/DAPI staining in $CD34^+$ and $CD34^-$ fractions of inv(16) AML samples transduced with shCtrl or shmiR-126.

(e) Frequency (%) of G_0, G_1 or $S/G_2/M$ phases of cell cycle in $CD34^-$ fraction of inv(16) AML samples (n=4) transduced with shCtrl (black) or shmiR-126 (red).

(f) Relative levels of *SPRED1* and *PLK2* in inv(16) AML $CD34^+$ cells transduced with shCtrl (black; n=4) or shmiR-126 (red; n=4), as assessed by qPCR and normalized using internal levels of *B2M*.

(g) Western blot of SPRED1, PLK2, p-ERK in $CD34^+$ cells from inv(16) AML patient samples (AML1298, AML987, AML1069) 2 days after transduction of shCtrl or shmiR-126.

(h) DNA fragmentation in inv(16) AML (AML1298, AML987, AML1069) $CD34^+$ cells 2 days after transduction of shCtrl or shmiR-126.

Each dot in (c), (e), (f) represents result from one AML patient sample; statistical significance of all comparisons was determined using two-tailed T tests (*$p< 0.05$; **$p<0.01$). Data are presented as the mean ± SD in (e), (f). Representative results of two experiments are shown in (g), (h).

Supplementary Figure 8

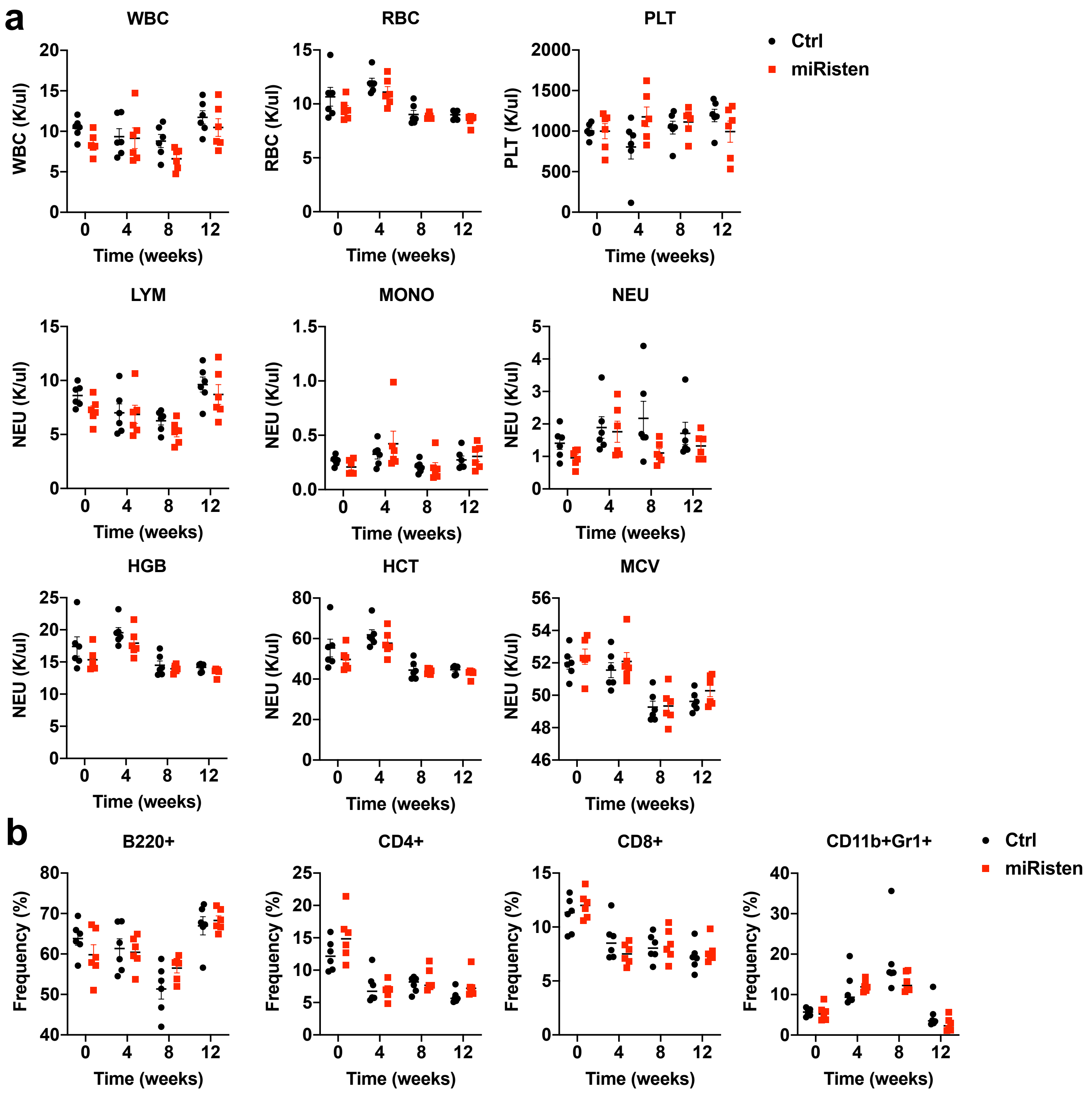

Supplementary Figure 8. Evaluation of effects of miRisten treatment on normal hematopoiesis.

(a) Complete blood count analysis of peripheral blood, white blood cells (WBC), red blood cells (RBC), platelets (PLT), lymphocytes (LYM), monocytes (MONO), neutrophils (NEU), hemoglobin (HGB), hematocrit (HCT), mean corpuscular volume (MCV) in PB from WT B6 mice at 0, 4, 8,12 weeks after treated with SCR (Ctrl; black) or miRisten (20 mg/kg/day, i.v., daily; red) for 3 weeks (n=6 mice for each group).

(b) Frequency of lineage populations in PB from WT B6 mice at 0, 4, 8,12 weeks after treated with SCR (black) or miRisten (20 mg/kg/day, i.v., daily; red) for 3 weeks (n=6 mice for each group). Each dot represents data from an individual mouse. Mean ± SEM are shown.

Supplementary Figure 9

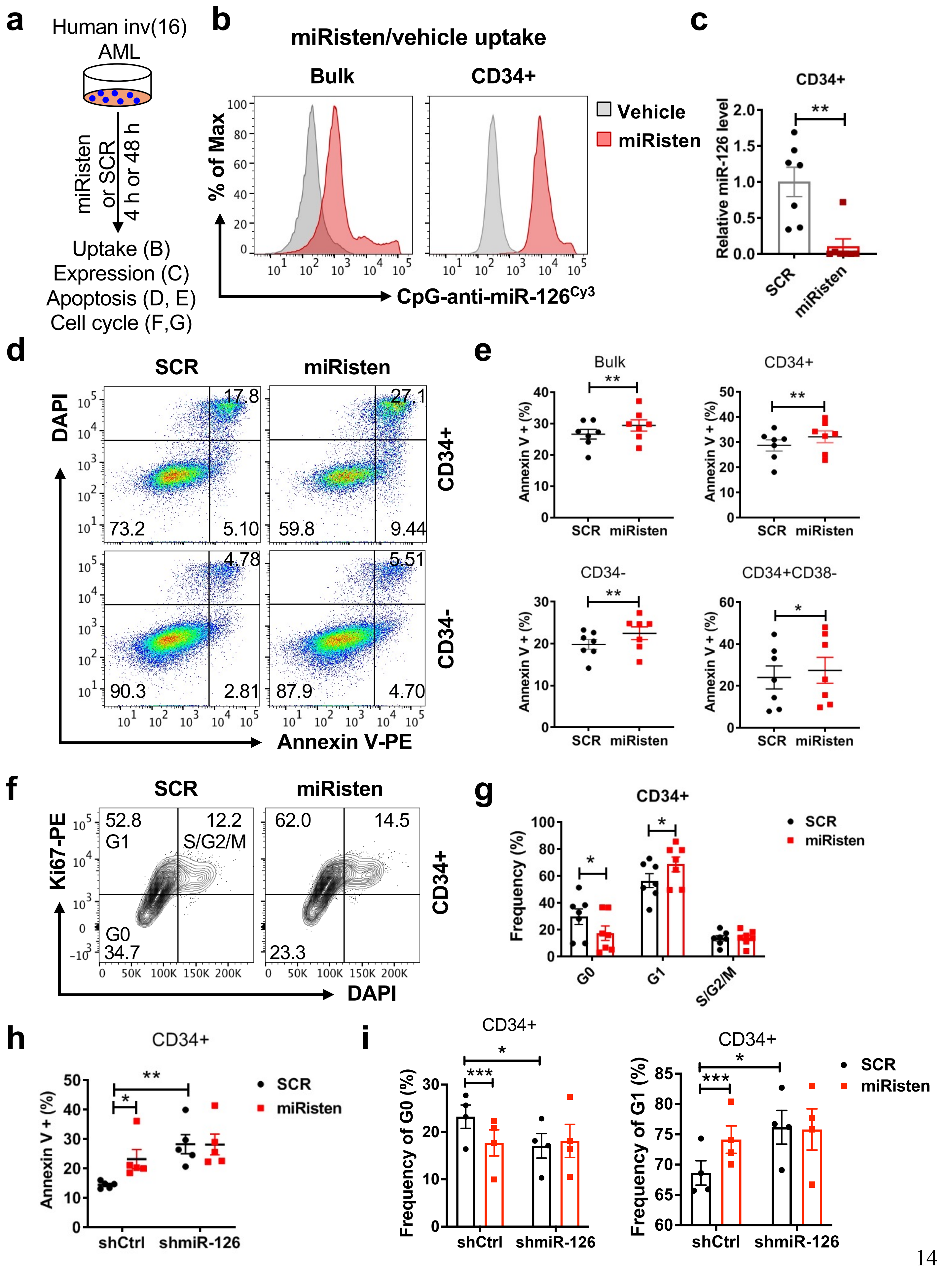

Supplementary Figure 9. Targeting miR-126 by miRisten treatment enhances apoptosis of inv(16) AML CD34$^+$ cells.

(a) Schematic of experimental design. Inv(16) AML cells were treated with miRisten or scramble control (1 µM) for 4h or 48h followed by analysis of update, miR-126 expression, apoptosis and cell cycle.

(b) The frequency (%) of uptake for miRisten (CpG-anti-miR-126^{cy3}; red) or vehicle (gray) in bulk or CD34$^+$ fractions of inv(16) samples.

(c) Relative levels of miR-126 in inv(16) CD34$^+$ fractions after treatment with SCR (black circle; n=7) vs. miRisten (1 µM; red square; n=7; p=0.0018), as assessed by qPCR and normalized by RNU44.

(d) Representative FACS plots showing gating strategy and frequency of Annexin V/DAPI staining in CD34$^+$ and CD34$^-$ fractions of inv(16) AML samples after treatment with SCR or miRisten (1 µM).

(e) Frequency (%) of Annexin V$^+$ apoptotic cells in SCR (black circle; n=7) vs. miRisten (1 µM; red square; n=7) treated inv(16) AML bulk (p=0.0055), CD34$^+$ (p=0.0063), CD34$^-$ (p=0.0013) and CD34$^+$CD38$^-$ (p=0.0233) fractions.

(f) Representative FACS plots showing gating strategy and frequency of Ki67/DAPI staining in CD34$^+$ fractions of inv(16) AML samples treated with SCR or miRisten (1 µM).

(g) Frequency (%) of SCR (black circle; n=7) vs. miRisten (1 µM; red square; n=7) treated inv(16) AML CD34$^+$ cells in G_0 (p=0.0128), G_1 (p=0.0137) or S/G_2/M phases of cell cycle.

(h) Frequency (%) of Annexin V$^+$ apoptotic cells in inv(16) AML CD34$^+$ cells transduced with shRNA control (shCtrl) vs. shmiR-126 (p=0.0031) and treated with SCR (black circle; n=5) vs. miRisten (1 µM; red square; n=5; p=0.029).

(i) Frequency (%) of inv(16) AML CD34$^+$ cells transduced with shCtrl vs. shmiR-126 (G_0 p=0.02; G_1 p=0.016) and treated with SCR (black circle; n=4) vs. miRisten (1 µM; red square; n=4) in G_0 (p=0.0005), G_1 (p=0.0009) phases of cell cycle.

Each dot represents result from an individual sample and data are presented as mean ± SEM. Statistical significance of all comparisons was determined using two-tailed student's T tests (*p< 0.05; **p< 0.01; ***p< 0.001).

Supplementary Figure 10

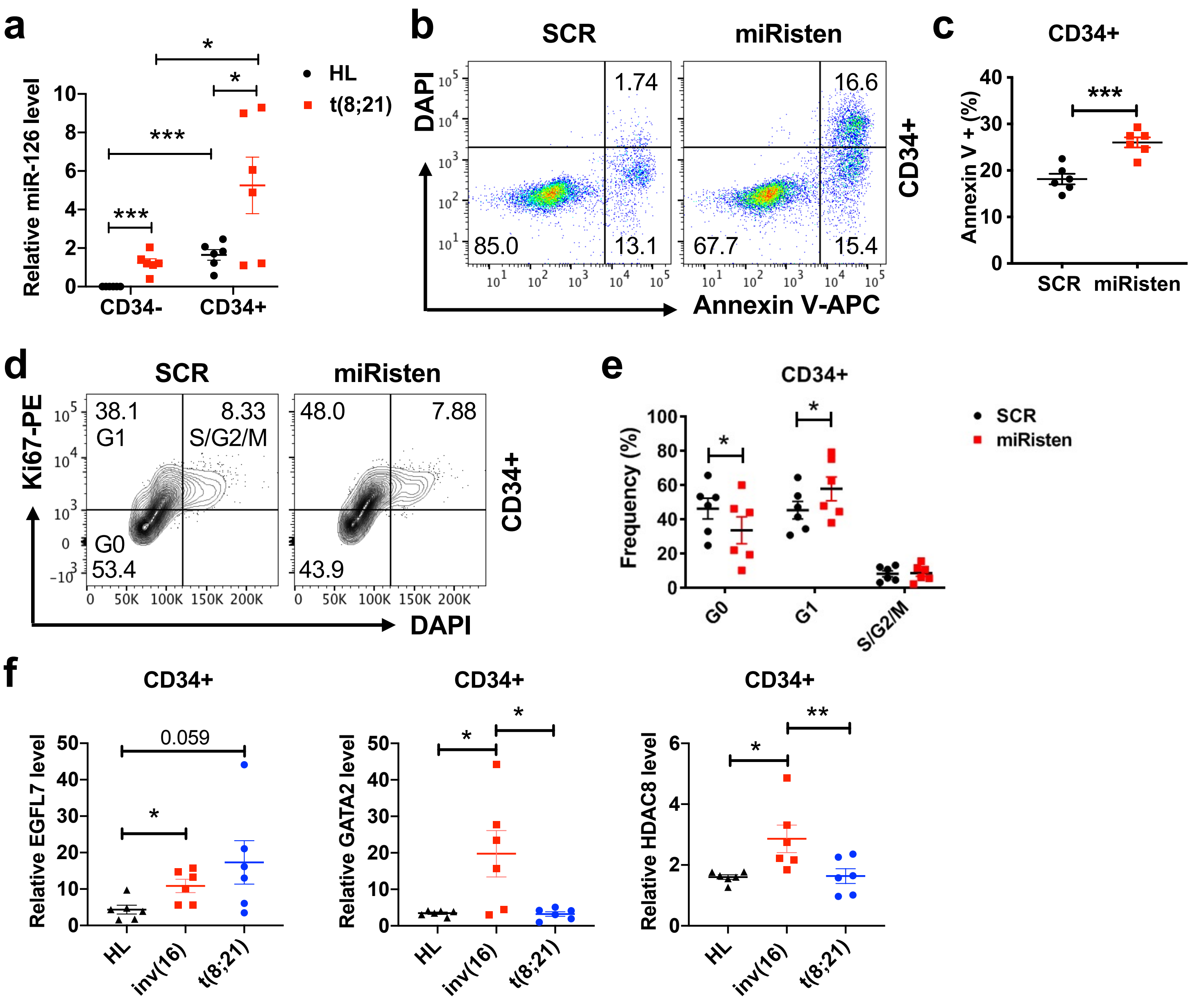

Supplementary Figure 10. Effect of miRisten treatment in t(8;21) AML cell survival and cell cycle. (a) Relative levels of miR-126 expression in $CD34^-$ and $CD34^+$ subsets from healthy (HL; black circle; n=6; HL $CD34^+$ vs. HL $CD34^-$ p=0.0001) donors and AML patients with t(8;21) (red square; n=6; t(8;21) $CD34^+$ vs. HL $CD34^+$ p=0.022; t(8;21) $CD34^-$ vs. HL $CD34^-$ p=0.0001; t(8;21) $CD34^+$ vs t(8;21) $CD34^-$ p=0.036) assessed by qPCR and normalized to RNU44.

(b) Representative FACS plots showing gating strategy and frequency of Annexin V/DAPI staining in $CD34^+$ fraction of t(8;21) samples after treatment with scramble (SCR) or miRisten (1 μM).

(c) Frequency (%) of apoptotic cells defined by Annexin V^+ in $CD34^+$ fraction of t(8;21) samples after treatment with SCR (black circle) vs. miRisten (1 μM; red square; n=6; p=0.0005).

(d) Representative FACS plots showing gating strategy and frequency of Ki67/DAPI staining in $CD34^+$ fraction of t(8;21) samples after treatment with SCR or miRisten (1 μM).

(e) Frequency (%) of t(8;21) AML $CD34^+$ treated with SCR (black circle; n=6) vs. miRisten (1 μM; red square; n=6) in G_0 (p=0.0144), G_1 (p=0.0132) or $S/G_2/M$ phases of cell cycle.

(f) Relative expression in $CD34^+$ cells from HL donors (black; n=6), inv(16) AML (red; n=6) or t(8;21) AML (blue; n=6) for *EGFL7* (HL vs. inv(16) p=0.0139; HL vs. t(8;21) p=0.059), *GATA2* (HL vs. inv(16) p=0.0283; inv(16) vs. t(8;21) p=0.0271) and *HDAC8* (HL vs. inv(16) p=0.0207; inv(16) vs. t(8;21) p=0.0382), assessed by qPCR and normalized to *B2M*.

Each dot represents data from an individual sample and data are shown as mean ± SEM. Significance for all comparisons were determined using two-tailed T tests (*p< 0.05; **p< 0.01; ***p< 0.001).

Supplementary Figure 11

a **b**

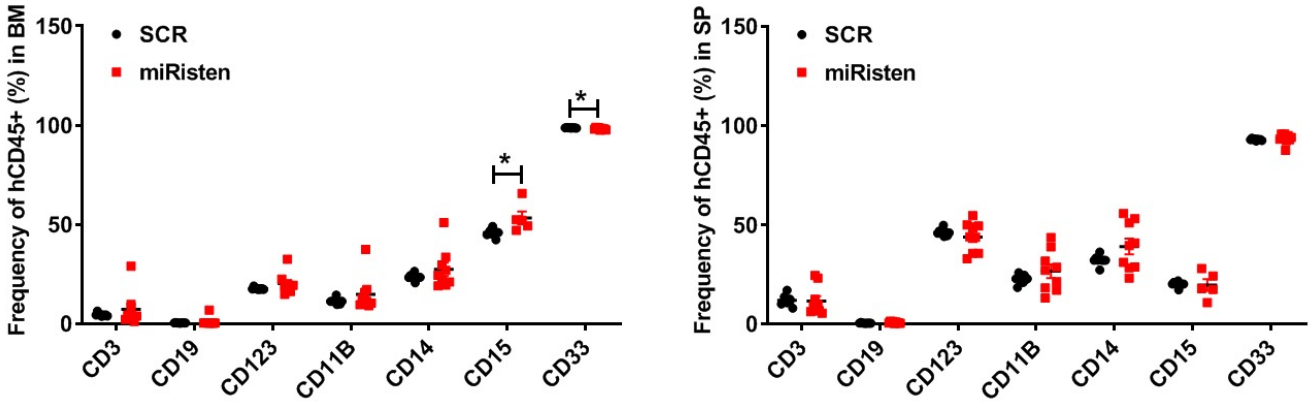

Supplementary Figure 11. Effects of miRisten treatment on immunophenotypic populations in inv(16) AML PDX.

(a) Frequency of immunophenotypic populations within $hCD45^+$ cells in BM of scramble (SCR) control (black circle) or miRisten (red square) treated primary mice (n=9; CD15 p=0.01; CD33 P=0.01 in BM).

(b) Frequency of immunophenotypic populations within $hCD45^+$ cells in SP of SCR control (black) or miRisten (red) treated primary mice (n=9).

Each dot represents data from an individual mouse. Data are shown as mean $\pm$ SEM. Significance of each comparison was determined using two-tailed T tests (*p< 0.05).

Supplementary Figure 12

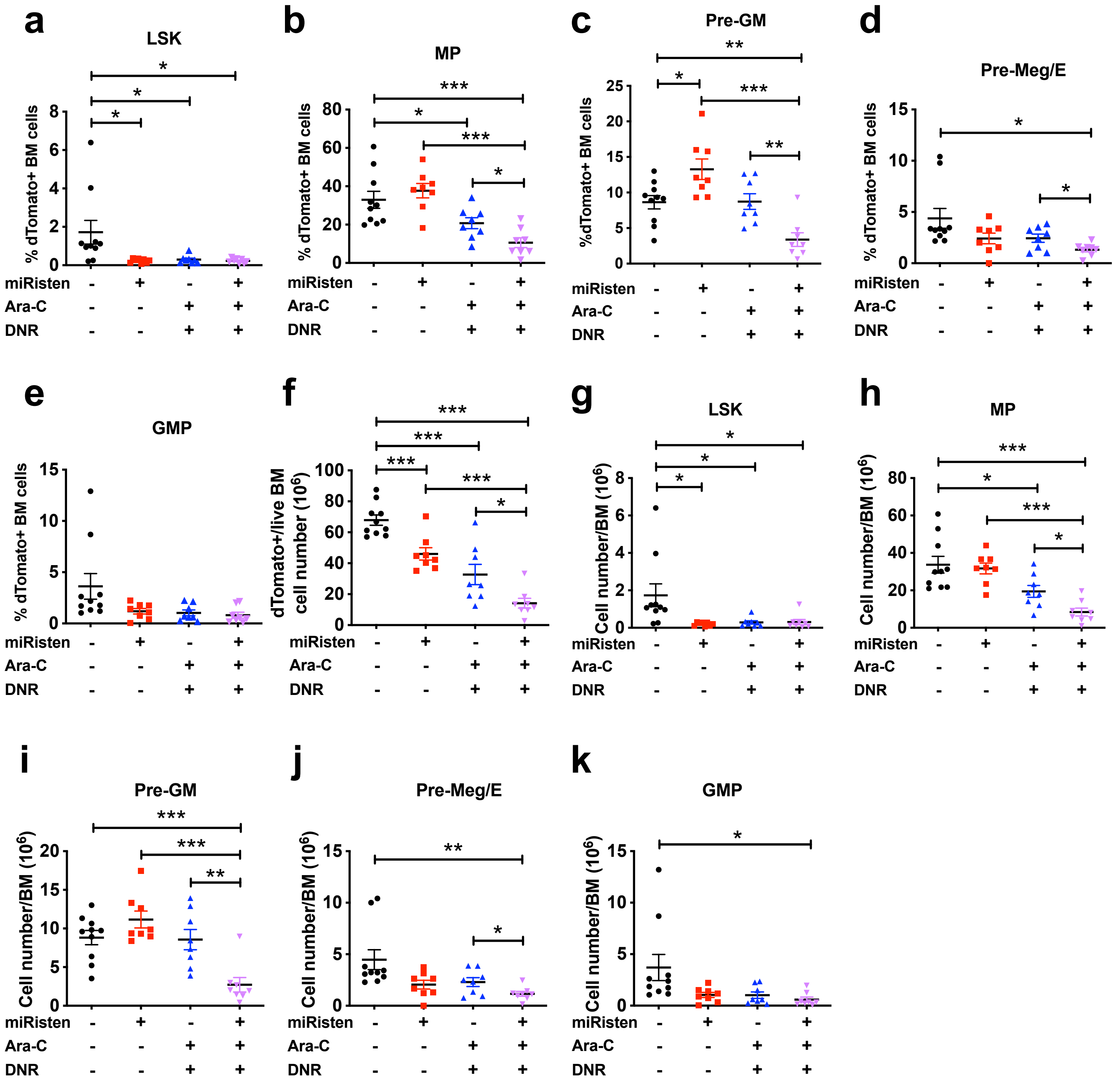

Supplementary Figure 12. Effects of miRisten treatment on CM-AML immunophenotypic subset.

(a) Frequency of dTomato+ AML cells in LSK within BM of mice treated with SCR control (black; n=10), miRisten (red; n=8; miRisten vs. SCR p=0.0322), A+D (blue; n=8; A+D vs. SCR p=0.0433), or miRisten/A+D (purple; n=8; miRisten/A+D vs. SCR p=0.0314).

(b) Frequency of dTomato+ AML cells in MP within BM of mice treated with SCR control (black; n=10), miRisten (red; n=8), A+D (blue; n=8; A+D vs. SCR p=0.0452), or miRisten/A+D (purple; n=8; miRisten/A+D vs. SCR p=0.0009; miRisten/A+D vs. miRisten p<0.0001; miRisten/A+D vs. A+D p=0.019).

(c) Frequency of dTomato+ AML cells in Pre-GM immunophenotypic subset within BM of mice treated with SCR control (black; n=10), miRisten (red; n=8; miRisten vs. SCR p=0.0129), A+D (blue; n=8), or miRisten/A+D (purple; n=8; miRisten/A+D vs. SCR p=0.0014; miRisten/A+D vs. miRisten p<0.0001; miRisten/A+D vs. A+D p=0.0026).

(d) Frequency of dTomato+ AML cells in Pre-Meg/E immunophenotypic subset within BM of mice treated with SCR control (black; n=10), miRisten (red; n=8), A+D (blue; n=8), or miRisten/A+D (purple; n=8; miRisten/A+D vs. SCR p=0.0135; miRisten/A+D vs. A+D p=0.0265).

(e) Frequency of dTomato+ AML cells in GMP immunophenotypic subset within BM of mice treated with SCR control (black; n=10), miRisten (red; n=8), A+D (blue; n=8), or miRisten/A+D (purple; n=8).

(f) Absolute cell number of dTomato+ AML cells in BM of mice treated with SCR control (black; n=10), miRisten (red; n=8; miRisten vs. SCR p=0.0007), A+D (blue; n=8; A+D vs. SCR p=0.0001), miRisten/A+D (purple; n=8; miRisten/A+D vs. SCR p<0.0001; miRisten/A+D vs. miRisten p<0.0001; miRisten/A+D vs. A+D p=0.0232).

(g) Absolute cell number of $dTomato^+$ LSK subsets in BM of mice treated with SCR control (black; n = 10), miRisten (red; n=8; miRisten vs. SCR p=0.0274), A+D (blue; n=8; A+D vs. SCR p=0.0412), miRisten/A+D (purple; n=8; miRisten/A+D vs. SCR p=0.0438).

(h) Absolute cell number of $dTomato^+$ MP in BM of mice treated with SCR control (black; n=10), miRisten (red; n=8), A+D (blue; n=8; A+D vs. SCR p=0.0247), miRisten/A+D (purple; n=8; miRisten/A+D vs. SCR p=0.0002; miRisten/A+D vs. miRisten p<0.0001; miRisten/A+D vs. A+D p=0.0122).

(i) Absolute cell number of $dTomato^+$ Pre-GM in BM of mice treated with SCR control (black; n=10), miRisten (red; n=8), A+D (blue; n=8), miRisten/A+D (purple; n=8; miRisten/A+D vs. SCR p=0.0003; miRisten/A+D vs. miRisten p<0.0001; miRisten/A+D vs. A+D p=0.0027).

(j) Absolute cell number of $dTomato^+$ Pre-Meg/E in BM of mice treated with SCR control (black; n=10), miRisten (red; n=8), A+D (blue; n=8), miRisten/A+D (purple; n=8; miRisten/A+D vs. SCR p=0.0087; miRisten/A+D vs. A+D p=0.0349).

(k) Absolute cell number of $dTomato^+$ GMP in BM of mice treated with SCR control (black; n=10), miRisten (red; n=8), A+D (blue; n=8), miRisten/A+D (purple; n=8; miRisten/A+D vs. SCR p=0.0464).

Each dot represent result from an individual mouse. Data are shown as mean $\pm$ SEM. Significance of each comparison was determined using two-tailed student's T test (*p< 0.05; **p< 0.01; ***p< 0.001).

Supplementary Table 1. Differentially expressed genes in CM/miR-126$^{\Delta/\Delta}$ LSK versus CM LSK

gene_id	logFC	logCPM	F	PValue	FDR	type
Marco	6.0590563	-2.440987	12.8594804	0.00365457	0.94538047	up
Capn8	3.91841474	-3.3289014	9.37169565	0.00838893	1	up
A930018P22Rik	3.67471613	-2.8192524	10.0566838	0.00525389	0.98773756	up
Ppara	3.28758261	-3.1494736	9.06428	0.00746872	1	up
Kdf1	3.17844762	-2.6435718	12.6734782	0.00221912	0.83836211	up
Mir693	3.06085619	-2.7162902	12.5499778	0.00230681	0.83836211	up
Insyn2b	2.61825004	-2.3824158	8.45034932	0.00935772	1	up
Olfr631	2.46587799	-2.3255921	11.8527153	0.00288074	0.88308794	up
Mir6400	2.45011571	-2.6917028	10.5038819	0.00450526	0.97399057	up
Mab21l1	2.24853993	-2.2139156	9.28144292	0.00690639	1	up
Ubqln5	2.17431165	-2.4647026	8.45750703	0.00933281	1	up
Lrrc69	2.06598831	-2.4643784	8.76986356	0.00831505	1	up
Mir1936	1.85382885	-1.7742962	11.8289206	0.00290297	0.88308794	up
Cldn5	1.80222474	-1.0272346	12.1081358	0.00265376	0.86429969	up
Kcnip1	1.79095093	-2.2127792	8.43801719	0.00940081	1	up
Tdrd9	1.76053511	-1.6384367	16.6026807	0.00070295	0.69300594	up
Naaladl2	1.75612137	-1.3441124	9.70162285	0.00594822	1	up
Pcdhb14	1.55272632	-1.5064068	12.9238892	0.00205253	0.83836211	up
9330175E14Rik	1.50859692	-0.9934074	8.65823238	0.00866372	1	up
9530091C08Rik	1.49102973	0.31484971	9.7625474	0.0063601	1	up
Il1rapl2	1.42811691	-0.7722432	9.12884268	0.00729634	1	up
C130026I21Rik	1.3988822	-1.6649987	8.45634699	0.00933684	1	up
Fbxw10	1.38185717	2.59947288	33.6521875	2.51E-05	0.08040169	up
Clec2f	1.34702767	0.7190506	22.559703	0.00015738	0.34026235	up
F420014N23Rik	1.33047935	-1.1724872	10.138505	0.00510708	0.98773756	up
Cyp26c1	1.2839376	-1.1479603	10.1048626	0.00516688	0.98773756	up
Mir1927	1.26253754	-0.6235392	10.2367044	0.00493693	0.98773756	up
Fam228a	1.25536191	-1.0722618	9.85118147	0.00564393	0.99393906	up
Mir6374	1.21530552	-1.0917139	8.49557442	0.00920158	1	up
Mir6368	1.20660909	-0.2956564	9.08031614	0.00742548	1	up
Serpinh1	1.16950292	0.7735524	19.8280751	0.00030293	0.45629098	up
Pcdhga9	1.16512774	2.31817874	12.3633395	0.0035521	0.94538047	up
Nbea	1.16410643	4.72610213	14.0977577	0.00335373	0.94538047	up
Pcdhgc5	1.13557844	0.25807325	9.86260265	0.00562142	0.99393906	up
Pcdhga4	1.11539558	0.58542756	13.6628155	0.001637	0.79209972	up
Pcdhga8	1.10753044	2.22320904	21.6067338	0.00020663	0.39859584	up
Pcdhgb8	1.09186391	1.80758585	10.8753794	0.00479578	0.98773756	up
Pcdhb18	1.07834046	0.36429833	9.93361379	0.0054837	0.99393906	up
Tmem269	1.04862205	-0.9761157	8.74354017	0.00839583	1	up
Pcdhgb2	1.04748025	1.06279723	13.5566928	0.00169044	0.79209972	up
Pcdhga11	1.03266211	1.95134789	18.4473998	0.00043009	0.56766943	up
Trim16	1.01115825	5.67389553	25.9559862	0.00030931	0.45629098	up
Gm16070	0.96728828	0.53927615	9.96830483	0.0054178	0.99393906	up
Pcdhga6	0.96380947	1.27679332	15.6667236	0.00091192	0.69300594	up
Pcdhga12	0.92152583	2.01849173	17.1844445	0.00060025	0.65447649	up
Neo1	0.91895277	1.11627387	12.4996351	0.00234366	0.83836211	up
Pcdhgb1	0.89762325	0.71731633	12.530163	0.00232123	0.83836211	up
Pcdhgb7	0.8961813	2.6447785	20.801047	0.00023857	0.39885161	up
Neb	0.88051897	2.44495568	9.06458601	0.00912354	1	up
Zfp286	0.87722239	3.27118942	66.2576443	1.82E-07	0.00228812	up
Pcdhga10	0.86031794	2.64173475	15.5952348	0.00101987	0.73075137	up
Pcdhgc4	0.84530539	1.13290597	10.2802379	0.00486354	0.98773756	up
Efcab7	0.77970348	2.64263284	14.7748878	0.00118545	0.77088266	up
Stxbp1	0.7560895	4.97500546	85.2754234	2.83E-08	0.00071023	up
Rftn2	0.71426058	2.79172641	11.1164661	0.00400298	0.94538047	up
Pcdhgb5	0.70443958	2.7773509	15.4883497	0.00095916	0.70746371	up
Pitx2	0.69411868	0.88908745	8.71650867	0.00847969	1	up
Etl4	0.69410588	2.39981127	13.6995429	0.00161895	0.79209972	up
Chil5	0.69003535	0.99318004	13.518167	0.00171031	0.79209972	up
Islr	0.68275742	2.21161763	14.7437832	0.00118808	0.77088266	up
Gipc3	0.68002499	1.91061672	9.08725733	0.00740685	1	up
F830016B08Rik	0.64731148	2.11039563	10.6344897	0.00430976	0.95940219	up
Pcdhga5	0.63456883	2.11972878	10.7732409	0.00411241	0.94615648	up
Fbn1	0.61293086	2.73797098	13.8977164	0.0015253	0.79209972	up
Slc27a4	0.60312522	4.66658092	51.5974101	1.06E-06	0.00882209	up
Gpr4	0.60102348	1.95804376	8.62743337	0.00876279	1	up
Pcdhga7	0.59912881	2.04381733	10.8030546	0.00407134	0.94538047	up
Synpo2	0.58612438	2.77527452	10.4449221	0.00459675	0.97692708	up
Slc24a3	-0.6288398	2.38221175	13.466851	0.0017372	0.79209972	down
Ubb	-0.6462034	7.14345048	14.883885	0.00224464	0.83836211	down
Ide	-0.6781496	8.06999933	40.8317178	6.67E-06	0.0418056	down
Rag2	-0.6908878	2.37226818	10.0874623	0.00519812	0.98773756	down
Podxl	-0.7318398	4.76232775	10.473719	0.00731973	1	down
Slc35d3	-0.7811069	2.95058691	12.7949567	0.00250453	0.83836211	down
Il4	-0.7996517	0.47495509	12.4093622	0.00241139	0.83836211	down
Lifr	-0.8104355	3.02873878	9.93904911	0.00715262	1	down
Siglech	-0.8852734	2.00130567	9.64268703	0.0066842	1	down
Gfra1	-0.9450802	1.08402632	8.70288408	0.00852232	1	down
Pltp	-1.0612858	0.8666145	14.5415586	0.00126037	0.77088266	down
Cadm1	-1.0984988	1.49339575	24.162779	0.00010948	0.27456449	down
St8sia1	-1.100601	2.17774849	18.0277313	0.00053482	0.60964448	down
Dlk1	-1.1006286	0.75562627	17.9895854	0.00048462	0.57872737	down
Cnnm1	-1.116509	-0.1995889	9.64558349	0.00606694	1	down
Ddr1	-1.1241288	0.11617891	10.0609784	0.00524607	0.98773756	down
Slc6a15	-1.1690317	2.64897469	12.2116885	0.00397843	0.94538047	down
Ccl24	-1.2554917	0.13545411	10.5894947	0.00437602	0.95940219	down
Dbx2	-1.5575525	-0.5727213	8.96752555	0.00958216	1	down
Cd209d	-1.5721562	-1.4083664	8.66904184	0.00862925	1	down
Ccdc92b	-1.7561665	-0.7821508	15.8555567	0.00086473	0.69300594	down
Pga5	-1.7848969	-1.6578617	8.82883072	0.00813726	1	down
Pax6	-1.7933246	-1.762043	12.6571488	0.00283299	0.88308794	down
Scn3a	-1.7974219	-1.3580686	8.88719579	0.00796548	1	down
Fgf15	-2.431621	-2.5194135	9.83517909	0.00567564	0.99393906	down
Efna5	-3.0889703	-2.5409188	9.45781586	0.00648454	1	down
Tph1	-4.1043805	1.26038948	20.4484695	0.00090612	0.69300594	down
Rnase2a	-4.3295838	-1.802338	14.9717205	0.00121757	0.77088266	down
Dlx4	-4.4052385	-3.0594539	12.5482522	0.00321203	0.93930513	down
Mir126a	-4.4097669	-3.0917927	12.0344237	0.00371772	0.94538047	down
Afm	-4.7760669	-2.776862	13.3532537	0.00257036	0.84815152	down
Asic4	-4.9840002	-2.9924714	11.111503	0.00657812	1	down

Supplementary Table 2. Top 13 most differentially enriched pathways in CM/miR-126$^{\Delta/\Delta}$ LSK versus CM LSK

Description	setSize	enrichmentScore	NES	pvalue	p.adjust	FDR	rank	leading_edge
HALLMARK_MITOTIC_SPINDLE	198	0.484503352	1.602957	0.015625	0.1039501	0.07878324	5247	tags=42%, list=21%, signal=33%
HALLMARK_MYC_TARGETS_V2	58	-0.773792205	-2.1552888	0.0212766	0.1039501	0.07878324	3270	tags=72%, list=13%, signal=63%
HALLMARK_REACTIVE_OXYGEN_SPECIES_PATHWAY	49	-0.578657132	-1.6249503	0.02272727	0.1039501	0.07878324	3896	tags=51%, list=16%, signal=43%
HALLMARK_UNFOLDED_PROTEIN_RESPONSE	112	-0.617500395	-2.0036114	0.02439024	0.1039501	0.07878324	2920	tags=42%, list=12%, signal=37%
HALLMARK_MYC_TARGETS_V1	198	-0.707392088	-2.4415438	0.02631579	0.1039501	0.07878324	4754	tags=67%, list=19%, signal=55%
HALLMARK_OXIDATIVE_PHOSPHORYLATION	197	-0.576570462	-1.9860463	0.02631579	0.1039501	0.07878324	6150	tags=62%, list=25%, signal=47%
HALLMARK_TNFA_SIGNALING_VIA_NFKB	200	-0.4885899	-1.6933572	0.02631579	0.1039501	0.07878324	4798	tags=48%, list=19%, signal=39%
HALLMARK_E2F_TARGETS	198	-0.420883232	-1.4526666	0.02631579	0.1039501	0.07878324	5377	tags=41%, list=21%, signal=32%
HALLMARK_P53_PATHWAY	197	-0.414736967	-1.4285969	0.02631579	0.1039501	0.07878324	5161	tags=40%, list=21%, signal=32%
HALLMARK_MTORC1_SIGNALING	199	-0.576219742	-1.9839419	0.02702703	0.1039501	0.07878324	5888	tags=60%, list=23%, signal=47%
HALLMARK_UV_RESPONSE_UP	161	-0.574794298	-1.9224873	0.02702703	0.1039501	0.07878324	4661	tags=48%, list=19%, signal=40%
HALLMARK_FATTY_ACID_METABOLISM	159	-0.497571687	-1.6584457	0.02702703	0.1039501	0.07878324	4420	tags=38%, list=18%, signal=32%
HALLMARK_ADIPOGENESIS	199	-0.412006461	-1.4185507	0.02702703	0.1039501	0.07878324	4386	tags=38%, list=17%, signal=32%

Supplementary Table 3. Characteristics of patient samples used in this study

Sample ID	FAB Classification	Sample Type	Disease Status	Cytogenetic	Other Mutation	Risk Status	Blasts In PB (%)	Blasts In BM (%)
AML1070	M4Eo	BM	Relapsed	der(16) inv(16) (p13.1q22) del(16) (q22.1q22.?2) [16]/[20] 44% CBFB rearrangement by FISH	NPM1 Neg,	Better-risk	3	30
AML021	M4	BM	Untreated	inv(16)		Better-risk	60	40
AML163	M4	PB	Relapsed	t(16;16), trisomy 21, trisomy 22	inv(16) Pos	Intermediate-risk	94	67
AML1298	M4	Leukocytes	Relapsed	t(16;16), Trisomy 22	FLT-3 ITD Neg., FLT-3 D835 Pos., NPM1 Neg., C-kit Neg	Poor-risk	76	
AML1260	M4	PB	Refactory	inv(16) (p13.1q22), +22 [20]	C-KIT Neg, FLT-3 Neg,	Intermediate-risk	61	
AML1069	M4Eo	PB	Relapsed	der(16) inv(16) (p13.1q22) del(16) (q22.1q22.?2) [16]/[20] 44% CBFB rearrangement by FISH	NPM1 Neg,	Better-risk	3	30
AML1260	M4	PB	Refactory	inv(16) (p13.1q22), +22 [20]	C-KIT Neg, FLT-3 Neg,	Intermediate-risk	61	
AML1327	M4	BM	Relapsed	inv(16) (p13.1q22), +22 [20]	C-KIT Neg, FLT-3 Neg,	Intermediate-risk	23	84
AML1241	M4eo	BM	Relapsed	inv(16),	C-KIT Neg,	Better-risk	2	
AML1363	M4	PB	Relapsed	inv(16)	NPM1 Neg., FLT3 ITD Neg., FLT3 D835 Neg.	Better-risk	61	
AML987	M4	PB	Relapsed	t(16;16), Trisomy 22	FLT-3 ITD Neg., FLT-3 D835 Pos., NPM1 Neg., C-KIT Neg.	Poor-risk	90	
AML1201		PB	Refractory	t(8;21),	Negative for CEBPA, KIT D816V, NPM1, FLT3 ITD & D835 variant mutations at diagnosis. Testing not repeated.	Better-risk	31	
AML113	M2	BM	Untreated	t(8;21); RUNX1/RUNX1T1	C-KIT Pos	Better-risk	50	95
AML107	M2	BM	Untreated	t(8;21), RUNX1/RUNX1T1		Better-risk	32	40
AML142		BM	Relapsed	t(8;21)		Better-risk	75	90
AML098	M2	PB	Relapsed	Variant t(8;21); RUNX 1/RUNX1T1		Better-risk	80	
AML099	M2	BM	Relapsed	Variant t(8;21); RUNX 1/RUNX1T1		Better-risk	80	

Supplementary Table 4. List of antibodies used for flow cytometry

No	Antibody name	Information
1	Biotin anti-mouse CD19, Clone: 6D5	1:400, Biolegend, 115504
2	Biotin anti-mouse NK1.1, Clone: PK136	1:400, Biolegend, 108704
3	Biotin anti-mouse TER-119, Clone: TER-119	1:200, Biolegend, 116204
4	Biotin anti-mouse/human CD45R/B220 Clone: RA3-6B2	1:200, Biolegend, 103204
5	Biotin anti-mouse IgM, Clone: RMM-1	1:200, Biolegend, 406504
6	Biotin anti-mouse CD3, Clone: 17A2	1:50, Biolegend, 100244
7	Biotin anti-mouse/human CD11b, Clone: M1/70	1:100, Biolegend, 101204
8	Biotin anti-mouse Ly-6G/Ly-6C (Gr-1), Clone: RB6-8C5	1:100, Biolegend, 108404
9	Biotin anti-mouse CD127 (IL-7Rα), Clone: A7R34	1:100, Biolegend, 135006
10	Biotin anti-mouse CD11c, Clone: N418	1:100, Biolegend, 117304
11	Biotin anti-mouse CD41, Clone: MWReg30	1:100, Biolegend, 133930
12	APC-eFluor 780 anti-mouse CD117 (c-kit), Clone: ACK2	1:100, eBioscience, 47-1172-82
13	Alexa Fluor 488 anti-mouse Ly-6A/E (Sca1), Clone: E13-161.7	1:100, Biolegend, 122516
14	Brilliant Violet 605 Streptavidin	1:100, Biolegend, 405229
15	Alexa Fluor 700 anti-Mo CD16/32, Clone: 93	1:100, eBioscience, 56-0161-82
16	PE/Cy7 anti-mouse CD105, Clone: MJ7/18	1:100, Biolegend, 120410
17	PE anti-mouse CD150, Clone: TC15-12F12.2	1:100, Biolegend, 133930
18	Pacific Blue anti-mouse CD48, Clone: HM48-1	1:100, Biolegend, 103418
19	APC anti-human CD45, Clone: 2D1	1:100, Biolegend, 368512
20	PE-Cyanline 7 CD34, Clone: 4H11	1:100, eBioscience, 25-0349-42
21	PE/Cy5 anti-human CD38, Clone: HIT2	1:100, Biolegend, 303508
22	PerCP/Cyanine 5.5 anti-human CD14, Clone: M5E2	1:100, Biolegend, 301824
23	eFluor 450 anti-human CD15, Clone: H198	1:100, eBioscience, 48-0159-42
24	FITC anti-human CD11B, Clone: ICRF44	1:100, Biolegend, 301330
25	Brilliant Violet 510 anti-human CD123, Clone:6H6	1:100, Biolegend, 306022
26	APC-eFluor 780 anti-human CD3, Clone:SK7	1:100, eBioscience, 47-0036-42
27	Alexa Fluor 700 anti-human CD19, Clone: SJ25C1	1:100, Biolegend, 363034
28	Brilliant Violet 605 anti-human CD33, Clone: P67.6	1:100, Biolegend, 366612
29	FITC Annexin V	5 µl/test, Biolegend, 640945
30	PE Annexin v	5 µl/test, Biolegend, 640947
31	APC Annexin v	5 µl/test, Biolegend, 640941
32	PE Mouse Anti-Ki-67, 51-36525X	20 µl/test, BD Biosciences, 55602

Supplementary Table 5. TaqMan gene expression assays used for qPCR analysis

No	Gene Symbol	Assay ID
1	Pri-miR-126	Mm03306244_pri
2	Pre-miR-126	Mm04335191_s1
3	miR-126	002228
4	Sno 234	001234
5	Egfl7	Mm00618004_m1
6	Gata2	Mm00492300_m1
7	Spred1	Mm01277511_m1
8	Plk2	Mm00446917_m1
9	E2f1	Mm00432939_m1
10	Cdk4	Mm00726334_s1
11	Npm1	Mm02391781_g1
12	B2m	Mm00437762_m1
13	EGFL7	Hs00211952_m1
14	B2M	Hs00187842_m1
15	RNU44	001094
16	GATA2	Hs00231119_m1
17	HDAC8	Hs00954353_g1
18	PLK2	Hs01573415_g1
19	BCL2	Hs04986394_s1
20	BAX	Hs00180269_m1
21	BAK1	Hs00832876_g1
22	SPRED1	Hs01084559_m1

Supplementary Table 6. Primer sequences used for qPCR analysis

Direction	Primer name	Sequence
Forward	*Cbfb-MYH11*	5’-GCAGGCAAGGTATACTTGAAGG-3’
Reverse	*Cbfb-MYH11*	5’-CTCTTCTCCTCATTCTGCTC-3’
Forward	*Hprt*	5’-TCCTCCTCAGACCGCTTTT-3’
Reverse	*Hprt*	5’-CCTGGTTCATCATCGCTAATC-3’
Forward	*A1-mCBF*	5’-CTAGAGGAGAGGTGGAGA-3’
Reverse	*A1-mCBF*	5’-CTGGCATAAATTTAGCTTGAGT-3’
Forward	*A2-mCBF*	5’-CCCCTCAGGCTCACAGAA-3’
Reverse	*A2-mCBF*	5’-GGGGAACAAGCATCTGTG-3’
Forward	*A3-mGATA2*	5’-GGGAGATGCAGCAGCCACAA-3’
Reverse	*A3-mGATA2*	5’-TGGAGACAGCCCCACTGACA-3’
Forward	*A4-mGATA2*	5’-GGGACTGAGGTCCAGGAGGG-3’
Reverse	*A4-mGATA2*	5’-GTGGCTGGCACCACTTCTCA-3’
Forward	*B1-mCBF*	5’-GTGGAGAAGAGAGTCTCTGA-3’
Reverse	*B1-mCBF*	5’-TCAGAGTGCCACCTCAGT-3’
Forward	*B2-mCBF*	5’-GAGATGGTGAATGGATACACTC-3’
Reverse	*B2-mCBF*	5’-TCTAGGAAACTAGTCATGAGGT-3’
Forward	*B3-mGATA2*	5’-GACCAGGGAGGCTCTGTCCA-3’
Reverse	*B3-mGATA2*	5’-GGCAGGAACCGCTGCAGTAT-3’
Forward	*B4-mGATA2*	5’-TAGGACAGAGAGGGGCAGGC-3’
Reverse	*B4-mGATA2*	5’-CTTGACCTCTGCTTCCGCCC-3’
Forward	*B5-mGATA2*	5’-ACACTGCAAGCTCAGCCTCG-3’
Reverse	*B5-mGATA2*	5’-CCCCTCCCTTACCCCCTAGC-3’
Forward	*hGATA2*	5’-GTTTGTCCAGGCCCCCTCAC-3’
Reverse	*hGATA2*	5’-CACAGGAGCCTCCCTTGCAG-3’
Forward	*hCBF/RUNX1*	5’-TGTTCTCCCTCTCCCCAGCC-3’
Reverse	*hCBF/RUNX1*	5’-CCCACCTCTCCTGCCTGAGT-3’

Supplementary Table 7. The sequences for siRNAs and shRNAs

No	Name	Sequence
1	siEbp1	5’-AUAAAGUUCUGGUGCGACA-3’<br>5’-GGUCUGAACUGAAUGAAGA-3’<br>5’-CCAAAGUGCUGCAGUACUA-3’<br>5’-GAGAAGAAAUGGAAGAAAU-3’
2	shHdac8-#1	CCGGGCCAGTGTTTAAAGTGTTTATCTCGAGATAAACACTTTAAACACTGGCTTTTTG
3	shHdac8-#2	CCGGCTACAGTGTCAATGTGCCCATCTCGAGATGGGCACATTGACACTGTAGTTTTTG
4	shGata2-#2	CCGGGGGCACCTGTTGTGCAAATTGCTCGAGCAATTTGCACAACAGGTGCCCTTTTTG
5	shGata2-#4	CCGGCCTGCAACACACCACCCGATACTCGAGTATCGGGTGGTGTGTTGCAGGTTTTTG

Supplementary Table 8. List of antibodies used for IP, IB and CHIP analysis

No	Antibody name	Information
1	Anti-CBFβ antibody	1:500, Santa Cruz Biotechnology, sc-56751
2	Anti-HSP90 antibody	1:2000, Santa Cruz Biotechnology, sc-13119
3	Anti-HDAC8 antibody	1:5000, Abcam, ab187139
4	Anti-β-actin antibody	1:2000, Sigma, A5316
5	Anti-Acetylated-Lysine antibody	1:1000, Cell Signaling Technology, 9681
6	Anti-Ebp1 antibody	1:1000, Bethyl Laboratories, A303-083A
7	Anti-Mdm2 antibody	1:500, Santa Cruz Biotechnology, sc-813
8	Anti-Ubiquitin antibody	1:2000, Millipore, 07-375
9	Anti-RAN antibody	1:1000, Santa Cruz Biotechnology, sc-1156
10	Anti-RCC1 antibody	1:1000, Santa Cruz Biotechnology, sc-1161
11	Anti-XPO5 antibody	1:1000, Cell Signaling Technology, 12565
12	Anti-SPRED1 antibody	1:2000, Abcam, ab64740
13	Anti-PLK2 antibody	1:1000, Cell Signaling Technology, 14812
14	Anti-p-ERK antibody	1:2000, Cell Signaling Technology, 4370
15	Anti-p-MYC(S62) antibody	1:1000, Cell Signaling Technology, 13748
16	Anti-MYC antibody	1:1000, Cell Signaling Technology, 5605
17	Anti-BCL2 antibody	1:1000, Cell Signaling Technology, 4223
18	Anti-BAX antibody	1:1000, Santa Cruz Biotechnology, sc-7480
19	Anti-PARP antibody	1:1000, Cell Signaling Technology, 9542
20	Anti-Cleaved Caspase 3 antibody	1:1000, Cell Signaling Technology, 9661
21	DYKDDDDK Tag (clone D6W5B) Rabbit monoclonal antibody	2 μg/ChIP, Cell Signaling Technology, 14793 Lot #5
22	CBFb Antibody - ChIP-seq Grade	4 μl/ChIP, Diagenode, C15310002
23	Recombinant Anti-RUNX1 / AML1 antibody	2 μg/ChIP, Abcam, ab272456
24	Anti-GATA2 antibody-ChIP Grade	2 μg/ChIP, Abcam, ab22849
