# Supplementary material for: Targeting miR-126 in inv(16) acute myeloid leukemia inhibits leukemia development and leukemia stem cell maintenance
Source: Nat Commun. 2021 Oct 22;12:6154. doi: 10.1038/s41467-021-26420-7 (PMC8536759; doi:10.1038/s41467-021-26420-7)
Supplement: Supplementary file 2 — Reporting Summary [file 41467_2021_26420_MOESM2_ESM.pdf]

nature research

Corresponding author(s): Ya-Huei Kuo

Last updated by author(s): Sep 17, 2021

Reporting Summary

Nature Research wishes to improve the reproducibility of the work that we publish. This form provides structure for consistency and transparency in reporting. For further information on Nature Research policies, see our Editorial Policies and the Editorial Policy Checklist.

Statistics

For all statistical analyses, confirm that the following items are present in the figure legend, table legend, main text, or Methods section.

n/a	Confirmed	
☐	☒	The exact sample size (n) for each experimental group/condition, given as a discrete number and unit of measurement
☐	☒	A statement on whether measurements were taken from distinct samples or whether the same sample was measured repeatedly
☐	☒	The statistical test(s) used AND whether they are one- or two-sided<br>*Only common tests should be described solely by name; describe more complex techniques in the Methods section.*
☒	☐	A description of all covariates tested
☐	☒	A description of any assumptions or corrections, such as tests of normality and adjustment for multiple comparisons
☐	☒	A full description of the statistical parameters including central tendency (e.g. means) or other basic estimates (e.g. regression coefficient) AND variation (e.g. standard deviation) or associated estimates of uncertainty (e.g. confidence intervals)
☐	☒	For null hypothesis testing, the test statistic (e.g. F, t, r) with confidence intervals, effect sizes, degrees of freedom and P value noted<br>*Give P values as exact values whenever suitable.*
☒	☐	For Bayesian analysis, information on the choice of priors and Markov chain Monte Carlo settings
☐	☒	For hierarchical and complex designs, identification of the appropriate level for tests and full reporting of outcomes
☒	☐	Estimates of effect sizes (e.g. Cohen's d, Pearson's r), indicating how they were calculated

Our web collection on statistics for biologists contains articles on many of the points above.

Software and code

Policy information about availability of computer code

Data collection	Graphs and statistical analysis was performed using Prism version 8.0 software (GraphPad Software). Flow cytometry analysis was performed using FlowJo software 10.6.1. The hierarchical clustering heatmap color scale is originated from the “redgreen” pallete in Cluster 3.0 software.
Data analysis	Raw small RNA sequences were trimmed to remove the 3’-adapter (TCTGGAATTCTCGGGTGCCAAGGAACTCC) using cutadapt v0.9.3. Reads longer than 16bp after trimming were aligned to mouse genome assembly mm9 using Bowtie v 0.12.7 with default settings. The expression level of mouse mature miRNAs from Sanger mirBase v18 were counted and the counts of miRNAs were then normalized by “TMM” method and counts per million (CPM) values were calculated by Bioconductor package “edgeR” v3.4.2. Raw RNA-seq sequences were subjected to adapter trimming using Trimmomatic v0.38 and poly(A) tails were removed using FASTP v0.19.4. The trimmed reads were aligned to mouse genome mm10 using Tophat v2.0.8 with default settings. Expression level of RefSeq gene (downloaded on 02/13/2020) were counted using HTSeq count v0.6.1. The raw count data were normalized by trimmed mean of M-values (TMM) method using Bioconductor package “edgeR” v3.20.9. Differential expression analysis was carried out using the quasi-likelihood (QL) F-test implemented in “edgeR” to determine the differentially expressed genes, with the cutoff of average RPKM in one group >= 1, p values <= 0.01 and fold change >= 1.5. The gene set enrichment analysis (GSEA) was performed by ClusterProfiler v3.10.1 to identify the affected GO, hallmark and KEGG pathways from MSigDB v7, using the pre-ranked gene list sorted by the -log10(p-value) with a sign determined by the fold change direction.

For manuscripts utilizing custom algorithms or software that are central to the research but not yet described in published literature, software must be made available to editors and reviewers. We strongly encourage code deposition in a community repository (e.g. GitHub). See the Nature Research guidelines for submitting code & software for further information.

Data

Policy information about availability of data

All manuscripts must include a data availability statement. This statement should provide the following information, where applicable:

- Accession codes, unique identifiers, or web links for publicly available datasets
- A list of figures that have associated raw data
- A description of any restrictions on data availability

Raw data for RNA-seq associated with Figure 1 was deposited GEO Accession: GSE133642. Raw microRNA-seq data associated with Figure 1 was deposited to GEO accession GSE173785. Raw data associated with Figure 3 was deposited to GEO accession number GSE184015. All data are available with no restriction.

Field-specific reporting

Please select the one below that is the best fit for your research. If you are not sure, read the appropriate sections before making your selection.

☒ Life sciences ☐ Behavioural & social sciences ☐ Ecological, evolutionary & environmental sciences

For a reference copy of the document with all sections, see nature.com/documents/nr-reporting-summary-flat.pdf

Life sciences study design

All studies must disclose on these points even when the disclosure is negative.

Sample size	Statistical analysis for normal distributions was performed using two-sample t-tests to analyze continuous variables. When a normal distribution was not satisfied, non-parametric Mann-Whitney U test was used. For categorical data, $\chi 2$ test or Fisher's exact test was used to compare proportions between groups. We estimated that 7 mice per group is required to obtain >80% power with a Type I error of 0.05 or a large effect size of 1.5. We adjusted the sample size based on detected effect size.
Data exclusions	No data was excluded from analysis.
Replication	All assays are conducted with replication in at least three independent experiments to confirm reproducibility.
Randomization	Simple randomization was performed to assign mice, or cell lines or patient samples were cultured in the same conditions and randomly divided into different treatment/conditions for experiments.
Blinding	During data collection and data analysis, the investigators used assigned sample ID and were not given grouping allocation information. Data analysis was performed and confirmed by multiple investigators.

Reporting for specific materials, systems and methods

We require information from authors about some types of materials, experimental systems and methods used in many studies. Here, indicate whether each material, system or method listed is relevant to your study. If you are not sure if a list item applies to your research, read the appropriate section before selecting a response.

Materials & experimental systems

n/a	Involved in the study	
☐	☒	Antibodies
☐	☒	Eukaryotic cell lines
☒	☐	Palaeontology and archaeology
☐	☒	Animals and other organisms
☐	☒	Human research participants
☒	☐	Clinical data
☒	☐	Dual use research of concern

Methods

n/a	Involved in the study	
☒	☐	ChIP-seq
☐	☒	Flow cytometry
☒	☐	MRI-based neuroimaging

Antibodies

Antibodies used	All antibodies names, manufacturer, number and dilution used in this study is detailed in Table S4 and S8.
Validation	Validation of all commercial antibodies for the species and specified application was stated by manufacturer and can be found on the manufacturer's website. Anti-HDAC8, anti-Ebp1 antibodies used in this study validated independently upon receipt by performing Western blot or immunoprecipitation using positive controls and genetic deleted (or knock-down) cells.

Eukaryotic cell lines

Policy information about cell lines

Cell line source(s)	32D clone 3 (ATCC CRL-11346) and 293T (CRL-3216) cells was purchased from the ATCC.
Authentication	Cell lines used in the studies were routinely monitored for changes in growth rate, cell morphology, cytokine dependency and characteristics of gene expression. However, they are not genetically authenticated.
Mycoplasma contamination	Mycoplasma testing were performed on cell lines every 3-6 months and to ensure no contamination was detected.
Commonly misidentified lines (See ICLAC register)	No commonly misidentified lines was used.

Animals and other organisms

Policy information about studies involving animals; ARRIVE guidelines recommended for reporting animal research

Laboratory animals	Cbfb56M/+, miR126flox/flox, Cbfb56M/+/Mx1-Cre, miR126flox/flox/Mx1-Cre, Cbfb56M/+/miR126flox/flox/Mx1-Cre, C57BL/6 and NOD/SCID/IL-2R-γ-/-/Tg (CMV-IL3, CSF2, KITLG) mice (6-8 weeks old), both males and females were used in the study.Mice were maintained on 12 hours light/12 hours dark cycles under 18-23°C ambient temperature with 40-60% humidity in an Association for Assessment and Accreditation of Laboratory Animal Care–accredited animal facility and all experimental procedures were performed in accordance with federal and state government guidelines and established institutional guidelines and protocols approved by the Institutional Animal Care and Use Committee at City of Hope.
Wild animals	This study did not involve wild animals.
Field-collected samples	This study did not involve field-collected samples.
Ethics oversight	All mice were maintained in an Association for Assessment and Accreditation of Laboratory Animal Care–accredited animal facility and all experimental procedures were performed in accordance with federal and state government guidelines and established institutional guidelines and protocols approved by the Institutional Animal Care and Use Committee at City of Hope.

Note that full information on the approval of the study protocol must also be provided in the manuscript.

Human research participants

Policy information about studies involving human research participants

Population characteristics	Human specimens were obtained from the Hematopoietic tissue bank at City of Hope. The covariants associated with each sample were not obtained as they were not relevant for this study.
Recruitment	Participants were recruited by the Hematopoietic tissue bank as part of a standard procedure with informed content and no potential bias.
Ethics oversight	This study was approved by the IRB committee at City of Hope.

Note that full information on the approval of the study protocol must also be provided in the manuscript.

Flow Cytometry

Plots

Confirm that:

- [x] The axis labels state the marker and fluorochrome used (e.g. CD4-FITC).
- [x] The axis scales are clearly visible. Include numbers along axes only for bottom left plot of group (a 'group' is an analysis of identical markers).
- [x] All plots are contour plots with outliers or pseudocolor plots.
- [x] A numerical value for number of cells or percentage (with statistics) is provided.

Methodology

Sample preparation	Samples were obtained from bone marrow or peripheral blood of mice or patients. Processing procedures are detailed in the method section or supplemental methods.
Instrument	Flow cytometry was performed using a 5-laser, BD LSRFortessa™ X-20 cell analyzer; sorting was performed on a 5-Laser, BD FACSAria Fusion Cell Sorter.
Software	Acquired data was analyzed by Flowjo software 10.6.1 with no customized code.

Cell population abundance	Post sorted populations were >95% pure based on flow cytometry.
Gating strategy	Preliminary gating for single cells were performed by FFC/SSC for all analysis. Examples of gating strategy is provided in the Figure (Figure 5D, 7B-C, 8D) or Supplementary Figure (S3A, S4B, S7D, S9D, S10B).

☒ Tick this box to confirm that a figure exemplifying the gating strategy is provided in the Supplementary Information.
